# The novel ORFV protein ORFV113 activates LPA-p38 signaling

**DOI:** 10.1371/journal.ppat.1009971

**Published:** 2021-10-06

**Authors:** Sushil Khatiwada, Gustavo Delhon, Sabal Chaulagain, Daniel L. Rock

**Affiliations:** 1 Department of Pathobiology, College of Veterinary Medicine, University of Illinois at Urbana-Champaign, Urbana, Illinois, United States of America; 2 School of Veterinary Medicine and Biomedical Sciences, Nebraska Center for Virology, University of Nebraska-Lincoln, Lincoln, Nebraska, United States of America; University of Otago, NEW ZEALAND

## Abstract

Viruses have evolved mechanisms to subvert critical cellular signaling pathways that regulate a wide range of cellular functions, including cell differentiation, proliferation and chemotaxis, and innate immune responses. Here, we describe a novel ORFV protein, ORFV113, that interacts with the G protein-coupled receptor Lysophosphatidic acid receptor 1 (LPA_1_). Consistent with its interaction with LPA_1_, ORFV113 enhances p38 kinase phosphorylation in ORFV infected cells *in vitro* and *in vivo*, and in cells transiently expressing ORFV113 or treated with soluble ORFV113. Infection of cells with virus lacking ORFV113 (OV-IA82Δ113) significantly decreased p38 phosphorylation and viral plaque size. Infection of cells with ORFV in the presence of a p38 kinase inhibitor markedly diminished ORFV replication, highlighting importance of p38 signaling during ORFV infection. ORFV113 enhancement of p38 activation was prevented in cells in which LPA_1_ expression was knocked down and in cells treated with LPA_1_ inhibitor. Infection of sheep with OV-IA82Δ113 led to a strikingly attenuated disease phenotype, indicating that ORFV113 is a major virulence determinant in the natural host. Notably, ORFV113 represents the first viral protein that modulates p38 signaling via interaction with LPA_1_ receptor.

## Author summary

Viruses have evolved mechanisms to subvert critical cellular signaling pathways that regulate diverse cellular functions. LPA signaling in skin is known to affect a wide range of cellular functions, including cell differentiation, migration, proliferation and chemotaxis, and it has been implicated in innate immune responses, inflammation and wound healing. Here, we show that ORFV113, a protein unique to parapoxviruses, interacts with the G protein coupled receptor, Lysophosphatidic acid receptor 1 (LPA_1_) enhancing p38 phosphorylation in ORFV-infected cells *in vitro* and keratinocytes *in vivo*, and in cells transiently expressing ORFV113 or treated with soluble ORFV113. p38 signaling markedly affects ORFV plaque formation and replication in infected cells. When ORFV113 is deleted from the viral genome, the resulting virus loses the ability to replicate to high titers and to induce gross lesions in the skin of sheep. Notably, ORFV113 represents the first viral protein that modulates p38 signaling by interacting with LPA_1_ receptor.

## Introduction

Orf or contagious ecthyma is a ubiquitous, highly contagious disease of sheep and goats caused by orf virus (ORFV). The disease is characterized by lesions at mucocutaneous margins in the mouth, reflecting virus tropism for keratinocytes [1]. Lesions progress from maculae to papulae, pustules and scabs, and usually resolve in 6–8 weeks [2]. The disease is usually mild and lesions remain confined to virus entry sites [3] and, although viremia has not been associated with ORFV infection, recently virus has been detected in blood of asymptomatic animals [4]. ORFV infection in sheep induces a potent early inflammatory response followed by a T-helper type 1 immune response at later stages. However, protective immunity against orf is short lived and virus can reinfect the host in both natural and experimental infections [5].

ORFV is the type member of the *Parapoxvirus* genus of *Poxviridae*, which also includes bovine pathogens pseudocowpox virus (PCPV) and Bovine papular stomatitis virus (BPSV), red deerpox virus, and Grey sealpox virus. The ORFV genome is approximately 138 kbp in length, and encodes for 132 genes [6]. The central genomic region contains genes highly conserved among the poxviruses that are essential for virus replication and structure, while the terminal genomic regions contains less conserved genes involved in virulence, and host range and immunomodulation [6]. The later genes are usually not essential for virus replication in cultured cells, but they affect virus virulence and pathogenesis in the host, and might play a role in viral persistence [7,8].

Based on sequence homology to host and viral genes, ORFV genes with putative immunomodulatory functions have been identified, including genes encoding IL-10-like protein (ORV127) [9], chemokine binding protein (ORV112) [10], vascular endothelial growth factor (VEGF) (ORV132) [11], granulocyte macrophage colony stimulating factor (GM-CSF) and IL-2-inhibitory factor or GIF (ORV117) [12], apoptosis inhibitor (ORV125) [13], and IFN resistance protein (ORV020) [14]. Five genes (ORFV-002, -024, -073, -119, and -121) were shown to encode novel NF-κB inhibitors [15–19].

Lysophosphatidic acid (LPA), is an ubiquitously expressed, short lived phospholipid mediator that signals through six different G-protein-coupled receptors (LPA_1_-LPA_6_) [20]. LPA signaling affects cell differentiation, proliferation, cytokine and chemokine secretion, cell migration and chemotaxis, and has been implicated in inflammation, cancer, wound healing and development [21–25]. LPA is produced either from lysophospholipids by the plasma enzyme, autotaxin (ATX/ENPP2) or from phosphatidic acid by activity of phospholipase A1 (PLA1) or phospholipase A2 (PLA2), and is found intra-and extracellularly [26–28]. LPA_1_, the most important of six LPA receptors, can bind to different G-protein α subunits, namely Gα12/13, Gαq11 and Gαi/o, to transduce Rho, Phospholipase C, MAP kinase (MAPK) and AKT signaling, respectively [20,23]. MAP kinases are part of the signaling cascade that respond to extracellular stimuli and comprises four discrete sub-groups, (1) extracellular signal-regulated kinases (ERKs), (2) c-jun N-terminal or stress-activated protein kinases (JNK/SAPK), (3) big MAP kinase 1 (BMK1), and (4) the p38 group of protein kinases [29]. p38 kinases consists of four different splice variants namely p38α, p38β, p38γ and p38δ. Of these, p38α and p38β are ubiquitously expressed while p38γ and p38δ are differentially expressed depending on the tissue type [30]. Treatment of cells with LPA induces upregulation of chemokines/cytokines such as interleukin-6 and interleukin-8 (IL-8) through activation of various signaling pathways including p38 MAPK, p42/44 MAPK, and Rho kinase [31]. In various cell types, including oral keratinocytes, LPA induced p38 MAPK activation leading to the transcription of IL-8 [32–35]. In rat aortic smooth muscle cells and human hepatocellular carcinoma (HCC) cell lines, LPA induces p38 activation leading to migration of cells and invasiveness, respectively [36,37].

Many viruses hijack G-protein-coupled receptors to ensure viral replicative success and often contribute to their pathogenesis. However, no direct role for LPA signaling in the context of virus infection has been reported. A recent study identified a pro-viral role of LPA signaling during Hepatitis C virus infection [38]. Hepatitis C virus replication was shown to induce expression of autotaxin (ATX) which activates LPA signaling by hydrolyzing lysophosphatidylcholine (LPC) to lysophosphatidic acid (LPA). Inhibition of ATX-LPA signaling decreased hepatitis C virus replication in hepatocytes.

While modulation of LPA signaling by viral factors has not been described, a few modulators of p38 signaling have been reported. For example, vaccinia virus protein A52R, known to inhibit IL-1- and Toll-like receptor-stimulated NF-κB activation, was found to activate p38 MAPK in a TRAF6 dependent manner [39,40]. E3, a vaccinia virus double-stranded RNA binding protein that inhibits cytokine expression, suppressed p38 phosphorylation in vaccinia virus-infected cells via an unknown mechanism [41].

Here, we show that ORFV113, a protein unique to parapoxviruses, enhances p38 phosphorylation in ORFV-infected cells *in vitro* and keratinocytes *in vivo*, and in cells transiently expressing ORFV113 or treated with soluble ORFV113. p38 signaling markedly affects ORFV plaque formation and replication in infected cells. We found that ORFV113 interacts with LPA_1_ receptor, which is involved in ORFV113-mediated enhancement of p38 activation. When ORFV113 is deleted from the viral genome, the resulting virus loses the ability to replicate to high titers and to induce gross lesions in the skin of sheep. Notably, ORFV113 represents the first viral protein that modulates p38 signaling by interacting with LPA_1_ receptor.

## Materials and methods

### Ethics statement

All animal procedures were approved by University of Nebraska-Lincoln Institutional Animal Care and Use Committee (IACUC; protocol 1796) and were performed in accordance with the Guide for the Care and Use of Agricultural Animals in Agricultural Research and Teaching.

### Cells and viruses

Primary ovine fetal turbinate (OFTu) cells were maintained in minimal essential medium (MEM) (Corning; 15010CV) supplemented with 10% fetal bovine serum (FBS) (Atlanta Biologicals, Flowery Branch, GA), 2 mM L-glutamine, gentamicin (50 μg/ml), penicillin (100 IU/ml), and streptomycin (100 μg/ml) [15]. Human foreskin fibroblast (HFF1) cells (obtained from American Type Culture Collection) were cultured in Iscove Dulbecco’s modified essential medium (IMDM) (Thermo Fischer Scientific; 31980030) supplemented as above. Cells were incubated at 37°C with 5% CO2. ORFV strain OV-IA82 [6] was used as a parental virus to construct ORFV113 gene deletion virus OV-IA82-Δ113 and for experiments involving wildtype virus infection. OV-IA82-Δ113 was used as parental virus to construct revertant viruses OV-IA82-RV113^Flag^ (ORFV113 with Flag tag), OV-IA82-RV113^HA^ (ORFV113 with HA tag) and OV-IA82-RV113 (untagged ORFV113).

### Plasmids

To construct expression plasmids pORFV113^Flag^, ORFV113 coding sequences were PCR-amplified from the OV-IA82 genome with primers 113-3xFlag-FW (*EcoRI*): 5’- TAAGGCCTCTGAATTCAATGGAAGTGTTGGTGATCGTCTC-3’;113-3xFlag-RV (*BamHI*): 5’-CAGAATACGTGGATCCCTACTCGTCGTAGAAAAACTCTCC-3’ and cloned into p3xFlag-CMV-10 vector (pFlag) and (Clontech, Mountain View, CA). Similarly, to obtain a ORFV113-HA (pORFV113^HA^) and untagged ORFV113 (pORFV113) expression vectors, *ORFV113* sequence was PCR amplified from OV-IA82 genome with primers 113-HA-FW (*EcoRI*): 5’-TAAGGCCTCTGAATTCGCCACCATGGAAGTGTTGGTGATCGTCTC-3’ and 113-HA-RV (*KpnI*): 5’-CAGAATACGTGGTACCCTCGTCGTAGAAAAACTCTCCCT-3’ and, UN113-FW (*EcoRI*): 5’-TAAGGCCTCTGAATTCGCCACCATGGAAGTGTTGGTGATC GTCTC-3’ and UN113-RV (*KpnI*): 5’-CAGAATACGTGGTACCCTACTCGTCGTAGAA AAACTCTCCCT-3’, and cloned into the vector pCMV-HA-C (Clontech, Mountain View, CA). DNA sequencing of plasmids confirmed the identity and integrity of the constructs.

### Construction of *ORFV113* gene deletion mutant and revertant viruses

To generate gene deletion mutant virus OV-IA82-Δ113, a recombination cassette (pΔ113-GFP) containing vaccinia virus 7.5 early-late promoter (VV7.5)-driven green fluorescent protein (GFP) gene flanked by *ORFV113* left and right flanking regions was constructed as previously described [42]. To generate revertant viruses OV-IA82-RV113^Flag^, sequences were synthesized containing C-terminally Flag tagged ORFV113 and a red fluorescent protein (RFP) reporter gene, all flanked by approximately 600 bp of homologous sequence on either side to mediate recombination (Genscript, Piscataway, NJ). To generate revertant viruses OV-IA82-RV113^HA^ (ORFV113 with HA tag) and OV-IA82-RV113 (untagged ORFV113), recombination plasmids containing ORFV113 sequence containing C-terminal HA tag or untagged ORFV113 and VV7.5-driven RFP reporter gene flanked by *ORFV113* left and right flanking regions were constructed.

To obtain OV-IA82-Δ113, OFTu cells were infected with OV-IA82 using a multiplicity of infection (MOI) of 1 and transfected with recombination plasmid pΔ113-GFP. To obtain the revertant viruses OV-IA82-RV113^Flag^, OV-IA82-RV113^HA^ and OV-IA82-RV113, OFTu cells were infected with OV-IA82-Δ113 (MOI, 1) and transfected with the respective recombination plasmids. Recombinant viruses were isolated by limiting dilution and plaque assay using fluorescence microscopy as previously described [16]. Identity and integrity of viral DNA sequences were confirmed by PCR and DNA sequencing.

### Virus purification and virion protein characterization

OFTu cells infected with OV-IA82, OV-IA82-Δ113, OV-IA82-RV113^Flag^, OV-IA82-RV113^HA^ or OV-IA82-RV113 (MOI, 0.1) were harvested at three days post infection (p.i.) and disrupted by three cycles of freeze and thaw. Cellular debris were removed by centrifugation at 1500 rpm for 5 min, and virus pelleted by ultracentrifugation at 25,000 rpm for 1 h. Pellets were resuspended in MEM, and pelleted again by ultracentrifugation, resuspended in MEM, aliquoted and frozen at -80°C. Viral titers were obtained in OFTu cells by the Spearman-Karber’s 50% tissue culture infectious dose (TCID_50_) method.

Intracellular mature virus (IMV) from OFTu cells infected with OV-IA82-Δ113 or OV-IA82-RV113^Flag^ was purified using double sucrose gradient protocol as previously described [19]. Whole cell extracts (50 μg) from OV-IA82-RV113^Flag^ infected OFTu cells, and purified IMV virions (10 μg) were resolved by SDS-PAGE, blotted to nitrocellulose membranes and probed with primary antibodies against Flag (Genscript; A00187), ORFV structural protein ORFV086 or host Glyceraldehyde-3-Phosphate Dehydrogenase (GAPDH) (Santa Cruz; sc-25778) overnight at 4°C [43]. Blots were incubated with the appropriate HRP-labeled secondary antibodies (1:15,000, anti-mouse, sc-2031 and anti-rabbit, sc-2004; Santa Cruz, respectively) and membranes were developed using chemiluminescent reagents (SuperSignal West Femto, Thermo Fischer Scientific).

### ORFV113 protein expression and subcellular localization

To evaluate ORFV113 expression during virus infection, OFTu cells were mock infected or infected with OV-IA82-RV113^Flag^ (MOI, 10) for 1, 2, 4, 6, 8, 12 or 24 h p.i. Total protein extracts (50 μg) were resolved by SDS-PAGE or native PAGE, blotted and transferred to nitrocellulose membranes, and probed with primary antibody against Flag and GAPDH. Blots were then incubated with appropriate HRP-labeled secondary antibodies and developed using chemiluminescent reagents.

The transcription kinetics of ORFV113 during ORFV infection in OFTu cells was examined by RT-PCR following procedures previously described [16]. Transcription of ORFV113, ORFV055 (late gene control) and ORFV119 (early gene control) was assessed by PCR using the primers 113intFw-5’-CGCCGTAATATGCTTAACCGGAGC-3’ and 113intRv-5’-CGGACCGTGTTGGTCGTTGGGTCT-3, 055LFw-5′-AATCATGGATCCGCCACCATGTTCTTCGCGCGTCGC-3′, and 055LRv-5′-TATCATCTCGAGCGGGCGTGGAGGTCGCCGAC C-3′ and 119intFw-5’-CTCTCGTAGGCTCGCTCTTG-3’ and 119EintRv-5’-GTCTCGAAGCA CCAGAGGTC-3’ respectively.

To assess the subcellular localization of ORFV113, OFTu cells (1.5 x 10^5^ cells/well) were cultured in four-well chamber slides (ibidi, Martinsried, Germany) for 16 h and mock infected or infected with OV-IA82-RV113^HA^ (MOI, 10). Cells were fixed at 1, 3, 6, 9, 12 or 24 h p.i. with 4% paraformaldehyde for 20 min, unpermeabilized or permeabilized with 0.2% Triton X 100 for 10 min, blocked with 1% bovine serum albumin (Sigma-Aldrich, St. Louis, MO) for 1 h, and incubated with primary antibody against HA overnight at 4°C. Cells were then incubated with Alexa fluor 488 labelled secondary antibody (Thermo Fisher Scientific; A-11005), counterstained with DAPI (2 μg/ml) for 10 min and visualized by confocal microscopy (A1, Nikon).

### Detection of ORFV113 in culture supernatants

To check for the presence of ORFV113 in supernatant of ORFV infected cultures, OFTu cells were mock infected or infected with OV-IA82-Δ113 or OV-IA82-RV113^HA^ (MOI, 10) and culture supernatants were harvested at 24 h p.i. Cellular debris and virions were removed from supernatants by centrifugation at 2000 rpm for 10 min and of the virus by ultracentrifugation at 25,000 rpm for 1 h, respectively. Immunoprecipitation (see below) was performed on clarified supernatants using HA antibody (Cell signaling; 3724) and immunoprecipitation products were resolved by SDS-PAGE, blotted and incubated with antibody against HA (Cell signaling; 2367). Blots were processed as described above.

The presence of ORFV113 was examined in supernatants of OFTu cells transfected with pCMV-HA (Empty HA plasmid), pCMV-ORFV113^HA^ (ORFV113^HA^ plasmid) or pCMV-ORFV113 (Untagged ORFV113 plasmid) (2 μg). Culture supernatants were harvested 24 h post transfection and clarified by centrifugation at 2000 rpm for 10 min. Fifty μl of clarified culture supernatants were resolved by SDS-PAGE, blotted and incubated with antibodies against HA.

### Growth curves

The replication characteristics of OV-IA82Δ113 was assessed in OFTu cells. Cells cultured in 12-well plates were infected with OV-IA82, OV-IA82Δ113 or OV-IA82-RV113^Flag^ using MOI 0.1 and 0.01 (multi-step growth curves) or 10 (single-step growth curve) and harvested at 6, 12, 24, 36, 48 and/or 60, 72, 84, 96 and 120 h p.i. Virus titers at each time point were determined as described above.

### Plaque assay

To assess the effect of ORFV113 on plaque formation, OFTu cells in 6-well plates were infected with OV-IA82Δ113 (GFP reporter) or OV-IA82-RV113^Flag^ (RFP reporter) (10^−5^ dilution of virus stock) for 1 h and cultures were washed three times with MEM media. Cultures were then overlaid with 2X MEM (MEM, 10% FBS, sodium bicarbonate, penicillin-streptomycin, Gentamycin and L-Glutamine) mixed with equal volume of 1% agarose. At 5 days post infection, random plaques (n = ~100) were imaged for each treatment and plaque area measured using Nikon A1 software.

### Co-immunoprecipitation

To prepare ORFV113-immunoprecipitation sample for mass spectrometry analysis of cellular interacting partners, OFTu cells was transfected with plasmid pCMV-ORFV113^Flag^ for 24 h and total cell protein was extracted as described above. Co-immunoprecipitation was performed using Active Motif Co-IP Kit (Active Motif, Carslbad, CA) following manufacturer’s protocols. Extracts were co-immunoprecipitated with anti-Flag antibody overnight at 4°C, and then incubated with 50 μl of pre-washed protein G agarose beads (Cat. #16–266; Millipore) at 4°C for 2 h. Beads were washed four times with high stringency buffer and bound proteins eluted in 1X Laemmli buffer. LC-MS Mass Spectrometry and data analysis were performed by Protein Sciences Facility, Roy J. Carver Biotechnology Center, UIUC. For data analysis, Xcalibur raw files were converted by Mascot Distiller into peak lists submitted to an in-house Mascot Server and searched against specific NCBI-NR protein databases, Thermo Proteome Discover, MaxQuant and GeneOntology modules.

To investigate the potential interaction of ORFV113 with LPA_1_, OFTu cells co-transfected with pcDNA3.1^His^ and pCMV^Flag^ or pCMV^HA^ (control plasmids) or pcDNA3.1-LPA_1_^His^ (pLPA_1_^His^) and pCMV-ORFV113^Flag^ (p113^Flag^) or pCMV-CD2v^HA^ (pCD2v^HA^) (plasmid with African swine fever virus membrane protein CD2v used as a specificity control) (1 μg) were harvested 24 h post transfection and membrane extracts were prepared using membrane protein extraction reagent (Thermo Fisher, Waltham, MA). Co-immunoprecipitation was performed on membrane protein extracts with antibodies against His (Genscript; A00186), Flag and HA (Cell Signaling Technology; 3724) as described above. Bound proteins were eluted from beads in 2X Laemmli buffer, resolved by SDS-PAGE, blotted to nitrocellulose membranes, probed with antibodies against His, Flag and HA (Cell Signaling Technology; 2367) and developed as described above.

### Co-localization of ORFV113 and LPA_1_

To investigate the cellular localization of ORFV113 and LPA_1_, OFTU cells mock infected or infected with OV-IA82RV113^HA^ for 24 h were fixed with 4% paraformaldehyde, sequentially incubated with primary antibodies against HA and LPA_1_ (Novus Biologicals; NBP1-03363), and secondary antibodies labeled with Alexa fluor 488 and 647, counterstained with DAPI, and examined by confocal microscopy.

### Effect of ORFV113 on p38 signaling in infected cells

To investigate the effect of ORFV113 on MAP kinase (MAPK) p38 signaling during ORFV infection. OFTu cells were mock-infected or infected with OV-IA82, OV-IA82-Δ113, OV-IA82-RV113^HA^ (ORFV113 with HA tag) or OV-IA82-RV113 (ORFV113 untagged) (MOI, 10) for 1, 3, 5, 12 or 24 h p.i. Total cell protein extracts were resolved by SDS-PAGE, blotted and incubated with antibodies against phos-p38 (Cell Signaling Technology; 4511) and total p38 (Cell Signaling Technology; 8690). Blots were processed as described above. Protein bands were quantified by densitometric analysis using ImageJ software, Version 1.6.0 (National Institute of Health, Bethesda, MD). Densitometry of phos-p38 bands were normalized to the loading control total p38.

### Effect of p38 inhibitor (SB203580) on ORFV replication and plaque formation

SB203580, a widely used p38 inhibitor, was selected to investigate the effect of p38 inhibition on ORFV replication and plaque size formation [44–46]. OFTu cells were pretreated with vehicle control (DMSO) or inhibitor SB203580 (10 μM) for 1 h and then infected with OV-IA82Δ113 (GFP reporter) or OV-IA82RV113 (RFP reporter). Plaque assays were performed in presence of DMSO or SB203580, and fluorescent plaques imaged at 5 days p.i.

To assess the effect of p38 inhibition on ORFV replication, OFTU cell pretreated with vehicle control (DMSO) or inhibitor SB203580 for 1 h were infected with OV-IA82 or OV-IA82Δ113 (MOI:0.1) in presence of DMSO or SB203580, and harvested at various time points, and virus titers determined in OFTu cells and expressed as TCID_50_/ml.

### Effect of ORFV113 expression on p38 phosphorylation

To investigate the effect of ORFV113 on p38 phosphorylation, HFF1 cells untreated, UV treated or transfected with pCMV-HA (Empty HA plasmid), or pCMV-ORFV113^HA^ (ORFV113^HA^ plasmid) (2 μg) were harvested at 2, 4 and 6 h post transfection. Total cell protein extracts (50 μg) were resolved by SDS-PAGE, blotted and incubated with antibodies against phos-p38, total p38, HA and GAPDH. Blots were processed and protein bands were quantified as described above.

To investigate the potential effect of ORFV113 present in cell culture supernatant on p38 signaling, OFTu cells were treated with clarified culture supernatants harvested from OFTu cells transfected with pCMV-HA (Empty HA plasmid), pCMV-ORFV113^HA^ (ORFV113^HA^ plasmid) or pCMV-ORFV113 (Untagged ORFV113 plasmid) for 3 h. Total cell extracts were prepared and p38 phosphorylation was assessed by Western blot and analysed as described above.

### Real-time PCR

To investigate the effect of ORFV113 on p38-regulated gene expression, OFTU and HFF1 cells were transfected with pCMV-HA (Empty HA plasmid) or pCMV-ORFV113^HA^ (ORFV113^HA^ plasmid) (2 μg) for 1, 2 and 3h and for 6h. RNA was extracted using Zymo Direct-Zol RNA extraction kit (Zymo, Cat # 74104) and reverse transcribed as previously described [15]. The expression of interleukin-8 (IL-8) gene was assessed using Custom Plus TaqMan Gene Expression Assays (Applied Biosystems) based on ovine gene sequences in GenBank. Real-time PCR were performed as previously described [15]. Experiments were conducted with biological and technical triplicates. Results were expressed as fold changes relative to empty HA plasmid treatment.

### Treatment of cells with immunoprecipitated ORFV113

OFTu cells transfected with pCMV-HA (Empty HA plasmid) or pCMV-ORFV113^HA^ (ORFV113^HA^ plasmid) (2 μg) were harvested 24 h post transfection and whole cell proteins were extracted using mammalian protein extraction reagent (Thermo Fisher, Waltham, MA). Immunoprecipitation was performed following manufacturer’s protocols. Whole cell extracts were added to 40 μl pre-washed anti-HA antibody conjugated agarose (Thermo Fisher; PI26181) and incubated overnight at 4°C. Beads were washed four times with 1X Tris buffered saline containing 0.05% Tween and protein was eluted with 130 μl anti-HA peptide (Thermo Fisher; PI26184). Presence of ORFV113^HA^ in immunoprecipitated product was assessed by western blot using antibody against HA as described above.

To investigate the effect of the ORFV113 protein on p38 phosphorylation, OFTu cells were treated with 60 μl of empty HA or ORFV113^HA^ immunoprecipitation product for 1 h and 3 h. Total cell protein extracts (50 μg) were resolved by SDS-PAGE, blotted and incubated with antibodies against phos-p38 and total p38. Blots were processed and protein bands were quantified as described above.

### Effect of siRNA LPA_1_ knockdown on ORFV113-induced p38 activation

To examine the role of LPA_1_ on ORFV113-induced phosphorylation, LPA_1_ expression was downregulated using siRNAs. OFTu cells were transfected with universal negative control siRNA (SIC001, Sigma Aldrich) or with three pooled siRNAs directed against sheep LPA_1_ 1-LPA_1_-(S): CCTACTTCTACCTAATGTT, LPA_1_-(AS): AACATTAGGTAGAAGTAGG; 2-LPA_1_-(S): CCCAATACTCGGAGACTGA, LPA_1_-(AS): TCAGTCTCCGAGTATTGGG; 3-LPA_1_-(S): GGTGGTTCTGTATGCTCAT, LPA_1_-(AS): ATGAGCATACAGAACCACC (Custom Oligos: Sigma Aldrich) (100 nM) for 24 h using Mission siRNA transfection reagent (S1452, Sigma Aldrich) and then either harvested for assessing LPA_1_ knockdown or treated with immunoprecipitation products from pCMV-HA or pORFV113^HA^ expressing cells and harvested at 1 h and 3 h post treatment. To measure the LPA_1_ knockdown, total cell protein extracts were resolved by SDS-PAGE, blotted and incubated with antibodies against LPA_1_ and GAPDH. Densitometry of LPA_1_ bands were normalized to the loading control GAPDH. Fold changes were calculated relative to universal negative control siRNA treatment. To assess the effect of LPA_1_ knockdown on ORFV113 induced p38 activation, total cell protein extracts were resolved by SDS-PAGE, blotted and incubated with antibodies against phos-p38 and total p38. Blots were processed and protein bands were quantified as described above.

### Effect of CRISPR LPA_1_ knockdown on ORFV113-induced p38 activation

To construct CRISPR LPA_1_ knockdown cells, OFTu cells were co-transfected with LPA_1_ CRISPR knock out plasmid and homology directed repair plasmid consisting of Puromycin drug resistance and RFP cassette (Santa Cruz) using Lipofectamine 2000 for 24h. Selection, amplification and maintenance of the culture were performed in the presence of Puromycin dihydrochloride (3μg/ml; Santa Cruz) and monitored by fluorescence microscopy. LPA_1_ knockdown was measured by western blot using LPA_1_ antibody (Santa Cruz; B-10).

To examine the effect of LPA_1_ knock down on ORFV113 induced p38 activation, wildtype and CRISPR knockdown OFTu cells cultured for 16 h were either harvested for assessing LPA_1_ knockdown or treated with immunoprecipitation products from pCMV-HA or pORFV113^HA^ expressing cells and harvested at 1 and 3 h post treatment. Level of LPA_1_ knockdown and effect of LPA_1_ knockdown on ORFV113 induced p38 activation were examined using western blot and densitometric analysis of bands as described above.

### Effect of antibody against LPA_1_ and LPA_1_ inhibitor (KI16425) on ORFV113 induced p38 signaling

To assess the effect of antibody against LPA_1_ on ORFV113 induced p38 phosphorylation, OFTu cells pretreated with IgG_2a_ mouse isotype control (Thermo Fisher; 026200) or LPA_1_ mouse monoclonal antibody (sc-515665;Santa cruz) (10 μg/ml) for 45 min were treated with immunoprecipitation products from pCMV-HA or pORFV113^HA^ expressing cells in presence of isotype control or LPA_1_ monoclonal antibody (10 μg/ml) and harvested at 1 h and 3 h post treatment.

KI16425, a widely used LPA_1_ inhibitor was used to investigate the role of LPA_1_ on ORFV113-induced p38 phosphorylation [47–49]. OFTu cells were pretreated with DMSO (vehicle control) or KI16425 (10 μM) for 45 min and were then treated with immunoprecipitation products from pCMV-HA or pORFV113^HA^ expressing cells in the presence of DMSO or KI16425 and harvested at 1 h and 3 h post treatment. Total cell protein extracts were resolved by SDS-PAGE, blotted and incubated with antibodies against phos-p38 and total p38. Blots were processed and protein bands were quantified as described above.

### Effect of LPA on ORFV113-induced p38 signaling

To investigate the effect of LPA on ORFV113-induced p38 signaling, phosphorylation of p38 was assessed by Western Blot. OFTu cells transfected with pEmpty^HA^ or pORFV113^HA^ plasmids for 5 h, were induced with LPA for 5, 10 and 20 min, and harvested. Total cell protein extracts (50 μg) were resolved by SDS-PAGE, and bands were blotted as above. Blots were incubated with antibodies against phos-p38, total p38 and HA. Blots were processed and protein bands were quantified as described above.

### Animal inoculations

To evaluate the effect of ORFV113 on ORFV virulence in the natural host, 66–80 lb. lambs were randomly allocated to three experimental groups, deletion mutant virus OV-IA82Δ113 (n = 4; sheep 5, 11, 55 and 130), revertant virus OV-IA82RV113^HA^ (n = 4; sheep 15, 40, 93 and 430) and mock (n = 3; sheep 25, 38 and 81). Following general anesthesia with ketamine/midazolam, the mucocutaneous junction of the lip near the right labial commissure and the inner sides of the hind limbs were scarified along 2 cm and 5 cm-long lines, respectively. Five hundred μl of virus inoculum (10^7.5^ TCID_50_/ml; virus groups) or PBS (control group) were then applied topically to each site using cotton swabs. The scarified areas of the lips were monitored for 20 days for the presence of characteristic orf lesions, including erythema, papules, pustules, and attached scab. Skin biopsy specimens were collected from hind limb inoculation sites at 1 day (d), 2 d, 3 d, and 7 d. p.i., fixed in 10% buffered formalin, and processed following standard histology procedures. To quantify the viral load, skin biopsies were frozen and thawed three times, sonicated, and viral titers determined as described above.

### Immunofluorescence in skin tissue sections

Antigen retrieval was performed on deparaffinized tissue sections using Citrate solution (Electron Microscopy Sciences; 50-192-6525) and following manufacturer’s instructions. Sections were permeabilized, blocked, incubated with primary antibody against HA (Cell Signaling Technology; 3724 and 2367), GFP (Thermo Fisher Scientific; A-11122 and A11120), LPA_1_ (Novus Biologicals; NBP1-03363), phos-p38 (Cell Signaling Technology; 4511) or IL-8 (Santa Cruz Biotechnology; sc-376750), and with appropriate Alexa fluor 488 and 647 labeled secondary antibodies, counterstained with DAPI, mounted with Prolong antifade reagent (Thermo Fisher Scientific; P10144) and examined by confocal microscopy.

### Accession numbers for PPV113 amino acid sequences

For sequence analysis, PPV113 amino acid sequences were aligned using Clustal Omega (EMBL-EBI). ORFV113 GeneBank accession numbers are (virus strains in parentheses) AAR98208.1 (OV-IA82), ABA00631.1 (NZ2), AHH34298.1 (B029), ADY76833.1 (D1701), NP_957890.1 (OV-SA00), AKU76735.1 (OV-GO), AKU76603.1 (OV-YX), AHZ33811.1 (NA1/11), AKU76867.1 (NP), AYM26054.1 (NA17), AYN61061.1 (SY17) and AKU76991.1 (OV-SJ1). PCPV113 and BPSV113 GeneBank accession numbers are ADC53885.1 (PCPV F00-120R), YP_003457420.1 (PCPV VR634) AEO18263.1 (PCPV IT1303/05), NP_958022.1 (BPSV BV-AR02), AKC03539.1 (BPSV BV-TX09c1) and AKC03281.1 (BPSV BV-TX09c15). Accession numbers for PPV113 of Parapoxvirus red deer strain HL953 and seal parapoxvirus strain AFK76s1 are YP_009112855.1 and YP_009389398.1, respectively.

### Statistical analysis

Data were presented as mean±standard deviation. Statistical analysis was performed using GraphPad Prism software Version 9.2.0. Statistical significance was determined by Student’s t-test for paired comparisons and by one-way ANOVA with post hoc Tukey test for multiple comparisons. A p< 0.05 was considered statistically significant.

## Results

### ORFV113 is a novel ORFV virion protein that is present at early and late times during infection and localizes in the cytoplasm and plasma membrane

*Parapoxvirus* 113 (PPV113) are conserved proteins not found outside the *Parapoxvirus* genus of the *Poxviridae*. Proteins are 186 to 238 amino acids in length, with % amino acid identities ranging from 37% to 97%. Comparison with available PPV113 sequences reveals that ORFV strain OV-IA82 ORFV113 shares 81%-97% amino acid identity with ORFV homologues isolated from sheep and goat, 64%-69% with PCPV, 36%-42% with BPSV, 47% with sealpox virus, and 38% with red deerpox virus. OV-IA82 *ORFV113* encodes for a protein of 211 amino acids, with predicted molecular weight of 22.2 kDa. ORFV113 lacks homology to known proteins outside the PPV genus, and domains suggestive of protein function. A N terminal transmembrane domain (IA82 positions 6–31) and two predicted N-linked glycosylation sites (IA82 positions 53 and 119) are present in the protein (**S1 Fig**).

**Fig 1:**
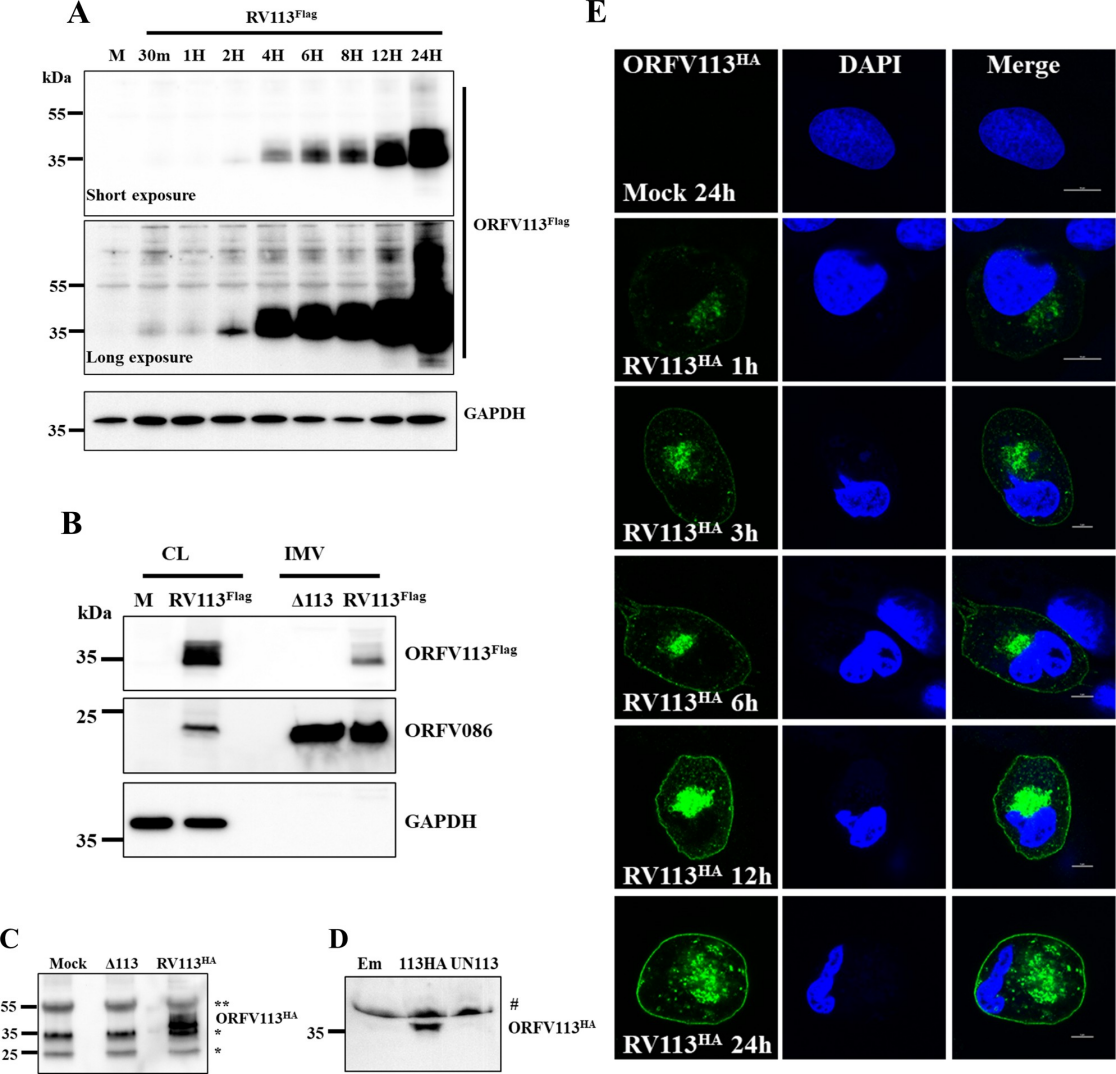
ORFV113 expression kinetics, presence in virions and supernatants, and subcellular localization. (A) Kinetics of ORFV113 expression. OFTu cells mock infected (M) or infected with revertant virus OV-IA82-RV113^Flag^ (MOI, 10) were harvested at indicated times p.i. Total cell protein extracts were resolved by SDS-PAGE, blotted and incubated with antibodies against Flag and GAPDH. Result is representative of two independent experiments. (B) Detection of ORFV113 in virions. OFTu cells were infected with OV-IA82-Δ113 (Δ113) or OV-IA82-RV113^Flag^ (RV113^Flag^), and intracellular mature virions (IMV) were purified through double sucrose gradient centrifugation. Whole cell lysate (CL) (50 μg) from OFTu cells mock infected or infected with OV-IA82-RV113^Flag^, and purified virions (10 μg) were resolved by SDS-PAGE and analyzed by Western blot using antibodies against Flag, structural protein ORFV086 or host GAPDH. Result is representative of three independent experiments. (C and D) Presence of ORFV113 in supernatant of ORFV infected cells and ORFV113 transiently expressing cells. (C) OFTu cells mock infected (M) or infected with OV-IA82-Δ113 or OV-IA82-RV113^HA^ (MOI, 10) were harvested at 24 h p.i. Immunoprecipitation was performed on clarified culture supernatant and immunoprecipitation products were resolved by SDS-PAGE, blotted and incubated with antibodies against HA. Result is representative of two independent experiments. (D) Culture supernatants were harvested from OFTu cells transfected with pCMV-HA (Empty HA plasmid), pCMV-ORFV113^HA^ (ORFV113^HA^ plasmid) or pCMV-ORFV113 (Untagged ORFV113 plasmid) at 24h post transfection. 50 μl of the culture supernatants were resolved by SDS-PAGE, blotted and incubated with antibodies against HA. Result is representative of three independent experiments. # indicates non-specific reactivity with serum protein routinely observed in supernatants. (E) ORFV113 subcellular localization. OFTu cells were mock infected or infected with OV-IA82-RV113^HA^ (MOI, 10), fixed at 1, 3, 6, 9, 12 and 24 h p.i. sequentially incubated with primary antibody against HA and Alexa fluor 488 labeled secondary antibody, counterstained with DAPI and examined by confocal microscopy.

The kinetics of ORFV113 expression was assessed during ORFV replication in primary ovine fetal turbinate cells (OFTu) by Western blot using a recombinant virus expressing C-terminally Flag-tagged ORFV113 (OV-IA82-RV113^Flag^). A protein of approximately 35 kDa was detected starting at 2 h p.i. with increasing protein levels and higher molecular weight species between 35 and 55 kDa observed at later time points (**Fig 1A**). The observed protein molecular weight was approximately 10 to 25 kDa higher than predicted, suggesting that the protein is post-translationally modified. Under non-reducing PAGE, ORFV113 species greater than 250 kDa were observed, suggesting non-covalent association with itself or other proteins under native conformation (**S2 Fig**). Consistent with the observation of early expression of ORFV113 by Western blot, ORFV113 transcription was detected early times during ORFV infection (1 to 2 h p.i.) (**S3 Fig**). ORFV113 transcript level were not affected in the presence of AraC, an inhibitor of DNA replication and of late poxviral gene transcription (**S3 Fig**). Together, these results indicate that ORFV113 belongs to the early class of poxviral genes.

The presence of ORFV113 in virions was investigated in intracellular mature virions (IMV) purified from OFTu cells infected with an ORFV113 gene deletion virus (OV-IA82-Δ113) or revertant virus expressing Flag tagged ORFV113 (OV-IA82-RV113^Flag^). Western blot analysis showed a predominant protein band (~35 kDa) and higher molecular weight species (~35 to 55 kDa) corresponding to ORFV113^Flag^ in OV-IA82-RV113^Flag^ virions but not in OV-IA82-Δ113 virions (**Fig 1B**). The virion core protein ORFV086 was used as a positive control and GAPDH was used as a control for potential cellular contamination. Data indicate that ORFV113 is a virion protein.

The presence of ORFV113 in supernatant of ORFV infected cells was investigated. OFTu cells were mock-infected or infected with OV-IA82-Δ113 or OV-IA82-RV113^HA^ for 24 h p.i., supernatants harvested, and immunoprecipitation and western blot performed using anti-HA antibody. ORFV113^HA^ was detected in the supernatant of OV-IA82-RV113^HA^-infected cells (**Fig 1C**). To check for the presence of ORFV113 in supernatant of cells transiently expressing ORFV113, OFTu cells were transfected with pCMV-HA (Empty HA plasmid) or pCMV-ORFV113^HA^ (ORFV113^HA^ plasmid) for 24 h, and supernatants harvested, and examined by western blot. Similar to ORFV infected cells supernatant, ORFV113^HA^ was detected in culture supernatant of cells transiently expressing ORFV113 (**Fig 1D**). Data indicate that ORFV113 is present in supernatant of ORFV infected cells and in cells transiently expressing ORFV113.

To examine the subcellular localization of ORFV113, OFTu cells were infected with OV-IA82-RV113^HA^ and examined by confocal microscopy at various times post-infection. ORFV113 staining was observed in paranuclear cytoplasmic region and plasma membrane starting at 3 h p. i. (**Fig 1E**). Thus, ORFV113 is present at early and late times in infected cells localizing in the cytoplasm and plasma membrane.

### ORFV113 is nonessential for ORFV replication in primary OFTu cells but affects ORFV plaque formation

**Fig 2:**
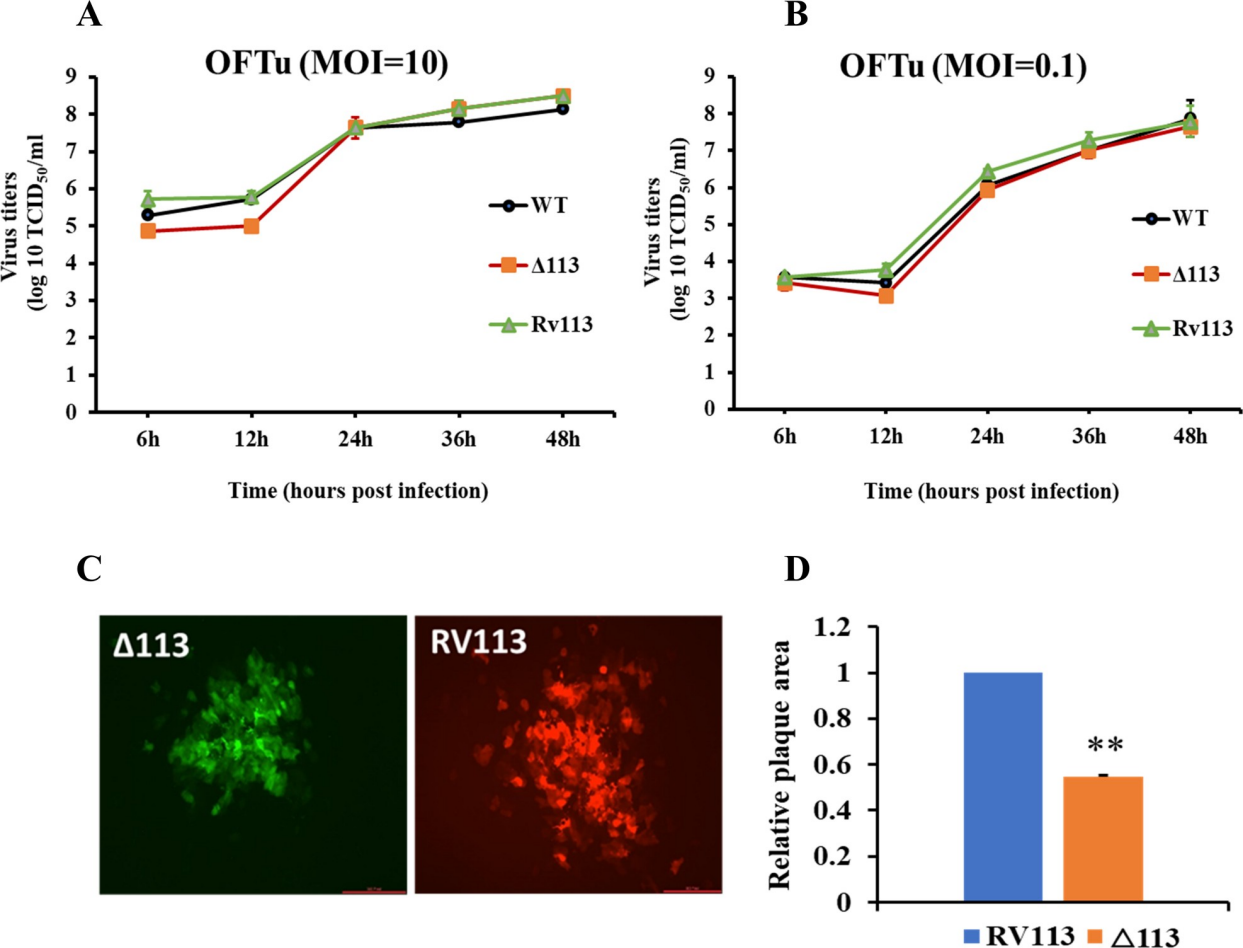
Replication characteristics of ORFV113-gene deletion virus. (A and B) OFTu cells were infected with wildtype OV-IA82 (WT), ORFV113 deletion mutant OV-IA82Δ113 (Δ113) or revertant OV-IA82RV113^Flag^ (RV113) viruses. (A) Single-step growth curve, MOI 10; (B) Multi-step growth curve, MOI 0.1. Titers were determined at indicated times p.i. and expressed as TCID_50_/ml. Results are average of two independent experiments. (C and D) Plaque morphology of ORFV113-gene deletion virus. OFTu cells were infected with OV-IA82Δ113 (Δ113) (GFP reporter) or OV-IA82RV113^Flag^ (RFP reporter) viruses and plaque assay was performed. Shown are representative fluorescent plaque images taken at 5 d p.i. (D) Random plaques were imaged (n = ~100), and plaque area was measured using Nikon A1 software. Relative Δ113 plaque areas compared to RV113 plaques are shown. Results are the average of three independent experiments. Error bars represent mean±SD. Statistical analysis was performed using paired Student’s t-test (**, *P<0*.*01*).

Replication kinetics of ORFV113 gene deletion virus (OV-IA82Δ113) was compared with that of revertant virus (OV-IA82-RV113^Flag^) in OFTu cells. No differences in replication kinetics and viral yields were observed in multi-step and single-step growth curves between the two viruses (**Figs 2A, 2B and S4**). Plaque assay showed OV-IA82Δ113 plaques were significantly reduced in size (approximately 45% smaller) when compared to OV-IA82-RV113^Flag^ plaques (**Fig 2C and 2D**). These data suggest that ORFV113 is nonessential for ORFV replication in OFTu cell cultures but affects ORFV plaque formation.

### ORFV113 interacts with Lysophosphatidic acid receptor 1(LPA_1_)

ORFV113 is a novel ORFV protein lacking homology with viral and host proteins in the gene bank and motifs suggestive of protein function. A preliminary mass spectrometry experiment using immunoprecipitates from cells transiently expressing ORFV113 identified Lysophosphatidic acid receptor 1, a MAPK p38 activator, as a putative ORFV113 interactor (Mascot score; 15, cut off score 3 at p<0.05) (**S5 Fig**) [32–35].

**Fig 3:**
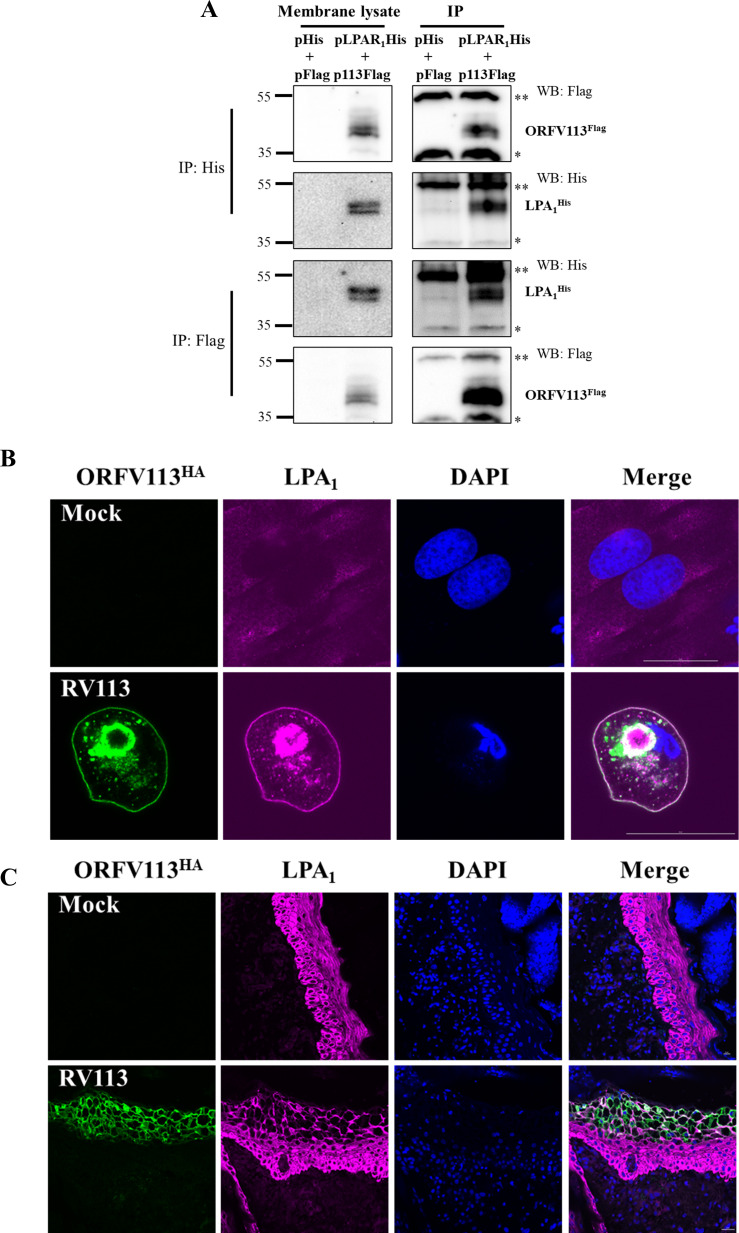
ORFV113 interacts with LPA_1_. (A) Co-immunoprecipitation of ORFV113 with LPA_1_. OFTu cells co-transfected with plasmids pcDNA3.1^His^ and pCMV^Flag^ (Empty plasmids) or pcDNA3.1-LPA_1_^His^ (pLPA_1_^His^) and pCMV-ORFV113^Flag^ (p113^Flag^) were harvested at 24 h post transfection and membrane fractions were extracted. Membrane lysates (left) and extracts immunoprecipitated with anti-His (upper panel) or anti-Flag (lower panel) antibodies were analyzed by SDS-PAGE-Western blotting with antibodies directed against proteins indicated on the right. * and ** denote light and heavy chain of the IgG antibody, respectively. (B) OFTU cells mock infected or infected with OV-IA82RV113^HA^ for 24 h were fixed and left unpermeabilized, and incubated with primary antibody against HA and LPA_1_, and Alexa Fluor 488 and 647 labeled secondary antibodies, counterstained with DAPI and examined by confocal microscopy. Green; ORFV113^HA^, Violet; LPA_1_, Blue; DAPI. (C) Skin sections from animals mock infected or infected with OV-IA82RV113^HA^ were processed for immunofluorescence and confocal microscopy as described above. Green; ORFV113^HA^, Violet; LPA_1_, Blue; DAPI.

To confirm the interaction between ORFV113 and LPA_1_, reciprocal co-immunoprecipitation experiments were performed. OFTu cells co-transfected with control plasmid or pORFV113^Flag^ together with plasmid expressing His tagged LPA_1_ (pLPA_1_^His^) were harvested at 24 h post-transfection and membrane proteins extracted. Reciprocal co-immunoprecipitation was observed following either anti-Flag or anti-His antibody pull downs (**Fig 3A**). As a control for specificity of ORFV113 and LPA_1_ interaction, CD2v^HA^ was used. No interaction was observed between CD2v^HA^ and LPA_1_^His^ in the membrane fraction of the cells expressing both CD2v^HA^ and LPA_1_^His^ (**S6 Fig**).

Colocalization of ORFV113 and endogenous LPA_1_ was assessed in ORFV infected cells. OFTu cells were mock infected or infected with OV-IA82-RV113^HA^ and fixed at 24h, immunofluorescence was performed using unpermeabilized cells. Colocalization of ORFV113 and LPA_1_ was observed in the membrane and paranuclear region of ORFV-infected cells (**Fig 3B**). Colocalization of ORFV113 and LPA_1_ also was examined in skin biopsies of ORFV infected and mock infected sheep. Colocalization of ORFV113 and LPA_1_ was observed in areas of ballooned degenerated keratinocytes in the outermost half of epidermis (**Fig 3C**). Together, data indicate that ORFV113 interacts with LPA_1_ *in vitro* and *in vivo*.

### ORFV113 enhances p38 phosphorylation in ORFV infected OFTu cells

**Fig 4:**
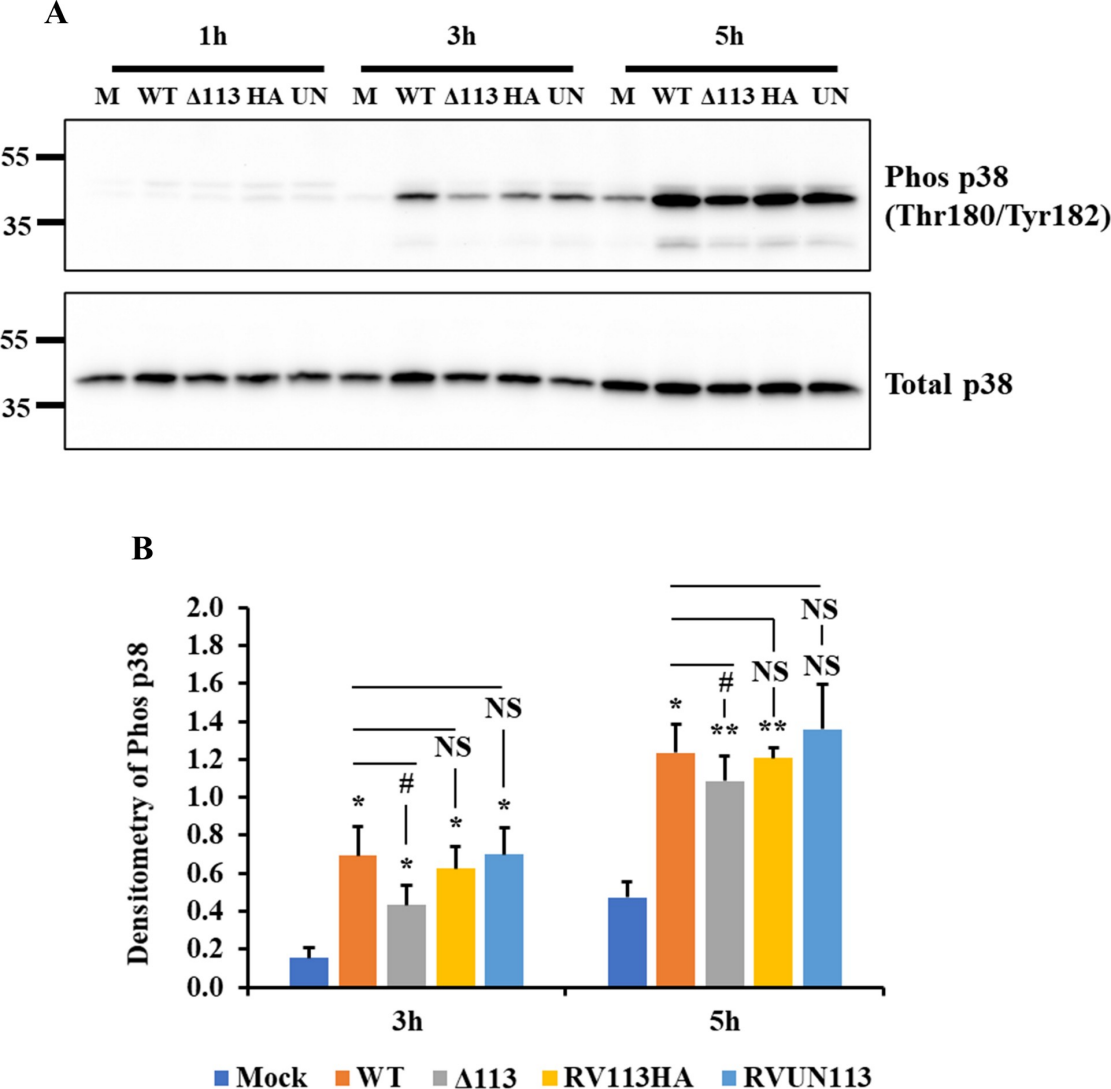
ORFV113 enhances p38 phosphorylation in infected cells. (A) OFTu cells were mock-infected or infected with OV-IA82 (WT), OV-IA82-Δ113 (Δ113), OV-IA82-RV113^HA^ (HA) and OV-IA82-RV113 (UN) (MOI:10) for 1, 3 and 5 h p.i. Total cell protein extracts were resolved by SDS-PAGE, blotted and incubated with antibodies against phos-p38 and total p38. (B) Densitometric analysis of bands corresponding to phosphorylated p38. Densitometry of phos-p38 bands were normalized to the loading control total p38. Results are average of three independent experiments. Error bars represent mean±SD. Statistical analysis was performed using one-way ANOVA with post hoc Tukey test for multiple comparisons (*,^#^, *P<0*.*05*; **, *P<0*.*01*; *NS*: *non-significant*). * and # denote statistical significance compared to mock and WT, respectively. Statistical comparison of a treatment to WT is shown with a line drawn over the respective treatment bar.

Activation of LPA signaling induces MAPK p38 phosphorylation in diverse cell types [32–35]. To investigate the effect of ORFV113 on p38 activation during ORFV infection, OFTu cells were mock-infected or infected with OV-IA82, OV-IA82-Δ113, OV-IA82-RV113^HA^ (HA-tagged revertant virus) or OV-IA82-RV113 (Untagged revertant virus) for 1, 3, 5, 12 and 24 h p.i. and phosphorylation of p38 was assessed by Western blot. Limited p38 phosphorylation was observed at 1 h p.i. and differences between the treatments were not evident (**Fig 4A**). Increased phosphorylation of p38 was observed at 3 and 5 h p.i. in virus infected cells compared to mock infected cells (**Fig 4A and 4B**). Notably, infection with OV-IA82-Δ113 led to significantly decreased p38 phosphorylation compared to OV-IA82 infected cells at 3 h (40% reduction) and 5 h (10% reduction) post infection and no significant difference was observed between OV-IA82 and both revertant viruses (**Fig 4A and 4B**). At 12 h p.i., significantly increased p38 phosphorylation was observed in virus infected cells compared to mock infected cells (**S7A and S7B Fig**). Although data trended toward reduced p38 phosphorylation in OV-IA82-Δ113 cells compared to OV-IA82 infected cells, significant differences were not observed (**S7B Fig**). At 24 h p.i., significantly increased p38 phosphorylation was observed in OV-IA82 and revertant virus infected cells compared to mock infected cells (**S7A and S7B Fig**). No significant difference was observed in level of p38 phosphorylation between OV-IA82 and OV-IA82-Δ113 infected cells (**S7B Fig**). Significant p38 phosphorylation was observed at 3, 5, and 12 h p.i. with OV-IA82-Δ113 infection compared to mock infected cells, suggesting that additional viral factors are involved in p38 phosphorylation in ORFV infected cells (**Figs 4A, 4B, S7A and S7B**). This shows that p38 signaling regulation during ORFV infection is complex, with ORFV113 possibly being one of several viral factors involved in temporal regulation of p38 signaling. Data indicate that ORFV113 enhances p38 phosphorylation at early times post ORFV infection.

### p38 signaling affects ORFV replication in infected OFTu cells

**Fig 5:**
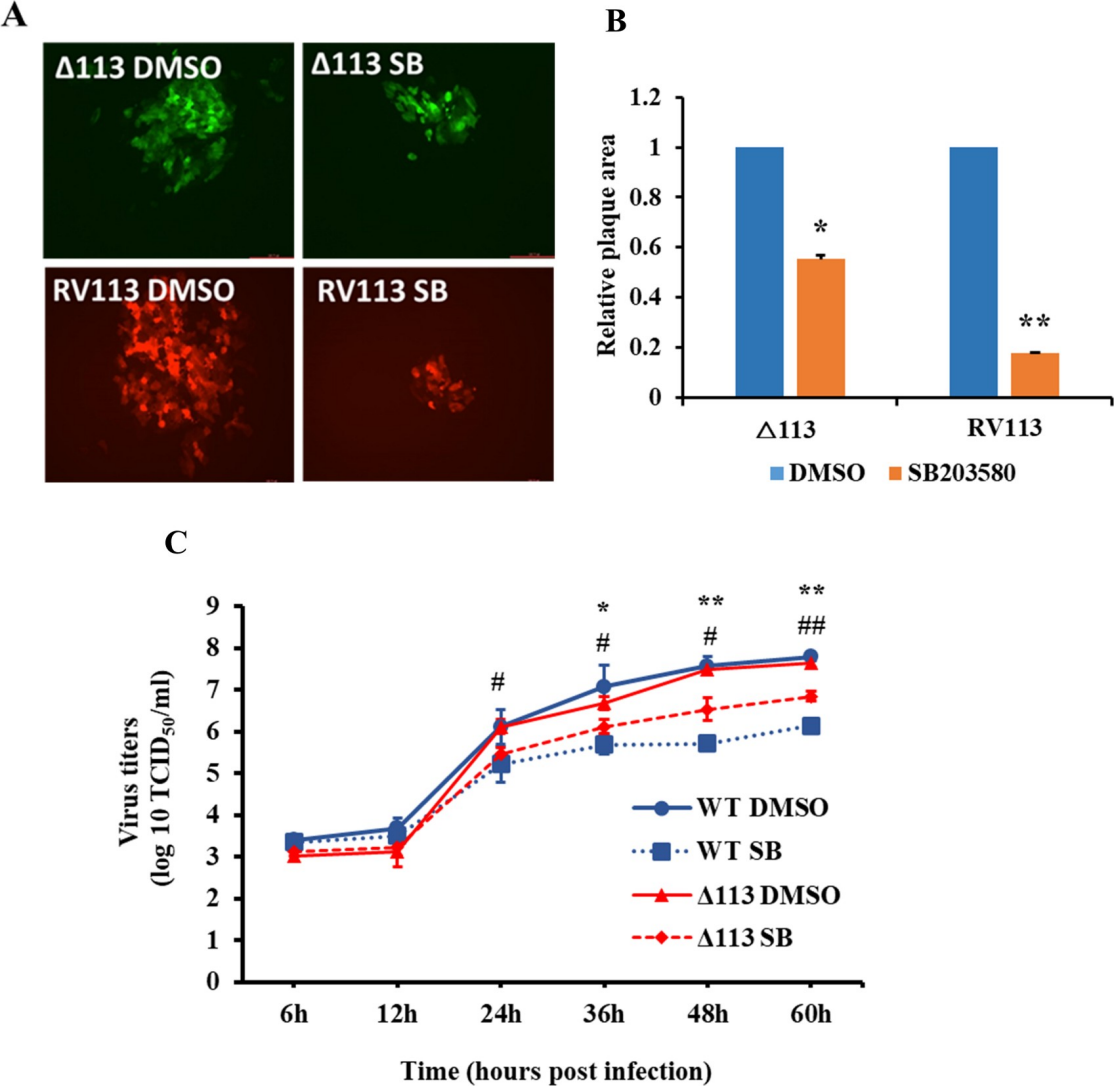
p38 signaling affects ORFV replication and plaque formation. (A and B) Effect of p38 inhibitor (SB203580) on ORFV plaque size. OFTU cell were pretreated with vehicle control (DMSO) or SB203580 for 1 h and infected with OV-IA82Δ113 (Δ113;GFP reporter) or OV-IA82RV113 (RV113;RFP reporter) and plaque assays were performed in presence of DMSO or SB203580. Shown are representative fluorescent plaque images taken at 5 days p.i. (B) Random plaques were imaged (n = ~100), and plaque areas measured using Nikon A1 software. p38 inhibitor treated Δ113 and RV113 plaques compared to DMSO treated Δ113 and RV113 plaques are shown. Results are average of two independent experiments. Error bars represent mean±SD. Statistical analysis was performed using paired Student’s t-test (*, *P<0*.*05*; **, *P<0*.*01*). (C) Effect of p38 inhibitor (SB203580) on ORFV replication, OFTU cell pretreated with vehicle control (DMSO) or SB203580 for 1 h were infected with OV-IA82 or OV-IA82Δ113 (MOI:0.1) in presence of DMSO or SB203580. Cultures were harvested at various time points and viral titers determined and expressed as TCID_50_/ml. Results are average of three independent experiments. Error bars represent mean±SD. Statistical analysis was performed using paired Student’s t-test (*, ^#^, *P<0*.*05*; **,^*#*#^, *P<0*.*01*). * and # denote statistical significance of DMSO OV-IA82 Vs SB203580 OV-IA82 and DMSO OV-IA82Δ113 Vs SB203580 OV-IA82Δ113, respectively.

To assess the importance of p38 signaling during ORFV infection, the effect of p38 inhibitor SB203580 on plaque size and ORFV replication was examined. OFTU cells were pretreated with vehicle control (DMSO) or SB203580 for 1 h and infected with OV-IA82-RV113^Flag^ or OV-IA82Δ113, and plaque assay performed in presence of DMSO or SB203580. Cells were fixed at 5 days post infection and fluorescent plaques were imaged (**Fig 5A**). OV-IA82-RV113^Flag^ plaques size was markedly reduced (82% area reduction) in presence of p38 inhibitor compared with plaques in presence of vehicle control. Significant reduction (45% reduction) was observed in OV-IA82Δ113 plaque size in presence of p38 inhibitor compared to vehicle control, however the effect was less remarkable than that observed for OV-IA82-RV113^Flag^ (**Fig 5B**).

To examine the effect of p38 inhibitor SB203580 on ORFV replication, OFTU cells pretreated with vehicle control (DMSO) or SB203580 for 1 h were infected with OV-IA82 or OV-IA82Δ113 in presence of DMSO or SB203580, harvested at various time points, and titrated. Wildtype virus titers were significantly reduced in presence of p38 inhibitor at 36h (1.5 log), 48h (1.8 log) and 72h (1.7 log) (**Fig 5C**). Significant reduction was also observed in OV-IA82Δ113 titers in presence of p38 inhibitor at 36h (0.5 log), 48h (1 log) and 72h (1 log), however, the effect was less dramatic compared to OV-IA82 (**Fig 5C**). Together the plaque assay and growth curve data in the presence of p38 inhibitor indicate that 1- p38 signaling increases ORFV yields, and 2-presence of ORFV113 enhances susceptibility of ORFV to p38 inhibitor effects.

### ORFV113 induces p38 phosphorylation in cell transiently expressing ORFV113 and in cells treated with soluble ORFV113 protein

**Fig 6:**
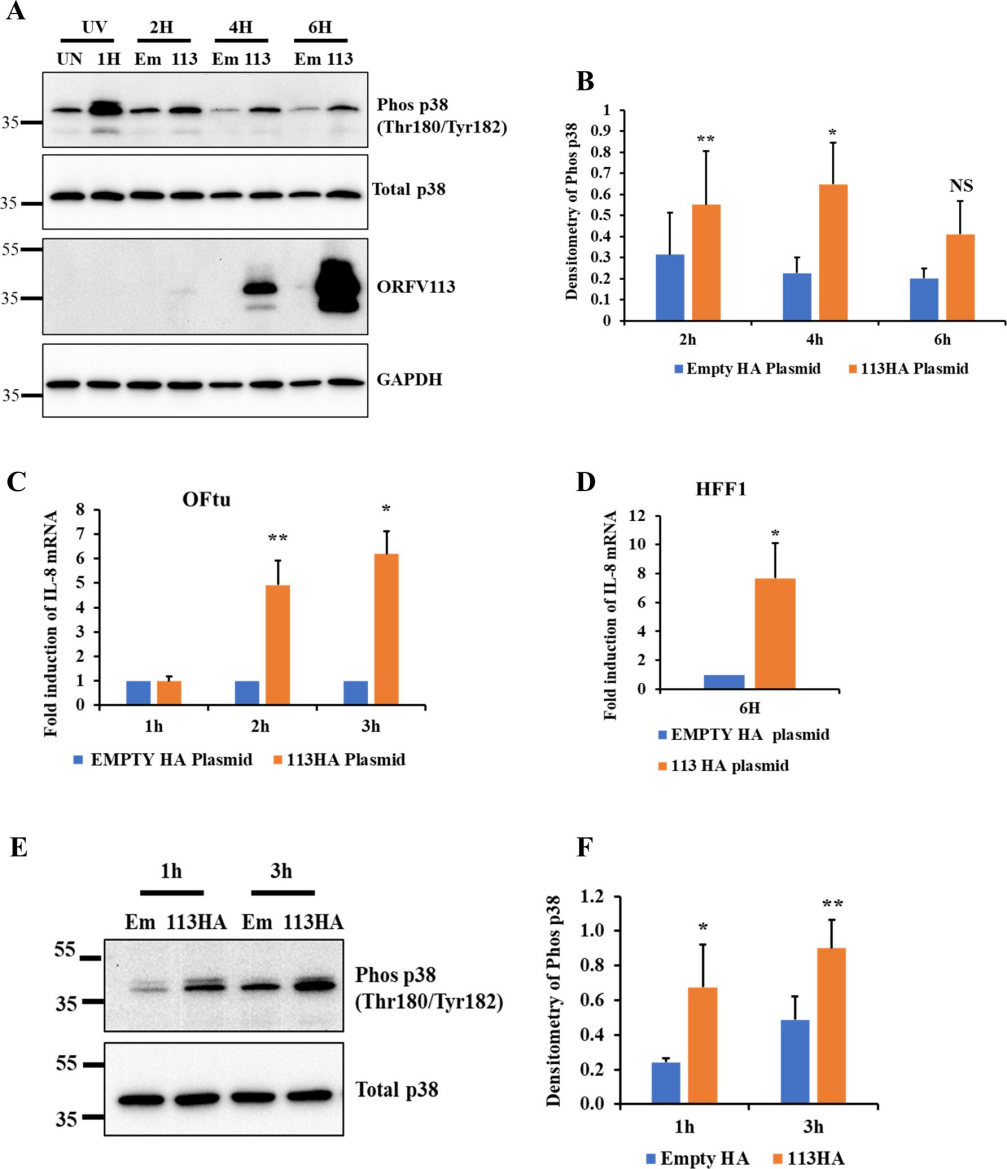
ORFV113 induces p38 phosphorylation and IL-8 transcription. (A) Expression of ORFV113 induces p38 phosphorylation. HFF1 cells untreated, UV treated or transfected with pCMV-HA (Empty HA plasmid) or pCMV-ORFV113^HA^ (ORFV113^HA^ plasmid) were harvested at respective time points. Total cell protein extracts were resolved by SDS-PAGE, blotted and incubated with antibodies against phos-p38, total p38, HA and GAPDH. (B) Densitometric analysis of bands corresponding to phosphorylated p38. Densitometry of phos-p38 bands were normalized to total p38 (loading control). Results are mean values of four independent experiments. Error bars represent mean±SD. Statistical analysis was performed using paired Student’s t-test (*P < 0.*05*). (C and D) Expression of ORFV113 induces IL-8 transcription. OFTU cells (C) were transfected with pCMV-HA or pCMV-ORFV113^HA^ for 1, 2 and 3 h and HFF1 cell (D) were transfected for 6h. Levels of IL-8 mRNA were assessed by real-time PCR. Each experiment was performed with biological and technical triplicates. Fold changes are relative to empty HA plasmid treatment. Results are the mean values of three independent experiments. Error bars represent mean±SD. Statistical analysis was performed using paired Student’s t-test (*, *P<0*.*05*; **, *P<0*.*01*). (E and F) Effect of soluble ORFV113 on p38 activation. (E) OFTu cells were treated with immunoprecipitation product from cells expressing pEmpty^HA^ or pORFV113^HA^ for 1 and 3 h post treatment. Total cell protein extracts were resolved by SDS-PAGE, blotted and incubated with antibodies against phos-p38 and total p38. (F) Densitometric analysis of bands corresponding to phosphorylated p38. Densitometry of phos-p38 bands were normalized to total p38. Results are the average of three independent experiments. Error bars represent mean±SD. Statistical analysis was performed as described in B (*, *P<0*.*05*; **, *P<0*.*01*).

To examine the possible effect of ORFV113 alone on p38 phosphorylation, human foreskin fibroblast (HFF-1) cells were untreated, treated with UV for 1 h (positive control), transfected with pCMV-HA (Empty HA plasmid) or plasmid pCMV-ORFV113^HA^ for 2, 4 and 6 h and p38 phosphorylation assessed by western blot. Significantly increased p38 phosphorylation was observed in cells expressing ORFV113 compared to empty plasmid transfected cells at 2h (1.8-fold) and 4h (2.9-fold) post transfection (**Fig 6A and 6B**).

To examine downstream gene expression regulated by p38 phosphorylation, OFTu and HFF-1 cells were transfected with pCMV-HA or pCMV-ORFV113^HA^, and IL-8 transcript levels assessed by RT-PCR. Significantly increased IL-8 transcription was observed in OFTu cells expressing ORFV113 compared to cells transfected with empty plasmid at 2h (4.9-fold) and 3h (6.1-fold) post transfection (**Fig 6C**) and at 6h (7.7-fold) post transfection in HFF1-cells expressing ORFV113 (**Fig 6D**). These data indicate that when transfected into cells ORFV113 induces p38 phosphorylation and activates downstream signaling leading to transcriptional activation of IL-8.

To investigate the potential effect of ORFV113 present in cell culture supernatant (**Fig 1C and 1D**) on p38 signaling, OFTu cells were treated with clarified culture supernatants harvested from OFTu cells transfected with pCMV-HA (Empty HA plasmid), pCMV-ORFV113^HA^ (ORFV113^HA^ plasmid) or pCMV-ORFV113 (Untagged ORFV113 plasmid) and phosphorylation of p38 was assessed by Western blot at 3 h post treatment. Treatment of cells with culture supernatants from cells expressing ORFV113^HA^ and Untagged ORFV113 induced significantly higher p38 phosphorylation (3.1 and 5.4 -fold, respectively) compared to control treatment (**S8 Fig**).

To rule out the effect of potential confounding cellular factors present in the culture supernatant on p38 signaling, OFTu cells were treated with immunoprecipitation product from cells expressing pCMV-HA (Empty HA plasmid) or pCMV-ORFV113^HA^ (ORFV113^HA^ plasmid) and phosphorylation of p38 assessed by Western blot at 1 and 3h post treatment. Treatment of cells with ORFV113 immunoprecipitation product induced significantly higher p38 phosphorylation (2.8 and 1.9-fold) compared to control treatment at 1 and 3 h, respectively (**Fig 6E and 6F**). Together, data indicate that both expression of *ORFV113* in cells and treatment of cells with soluble ORFV113 protein induces p38 activation.

### LPA_1_ is involved in ORFV113 induced p38 phosphorylation in OFTu cells

**Fig 7:**
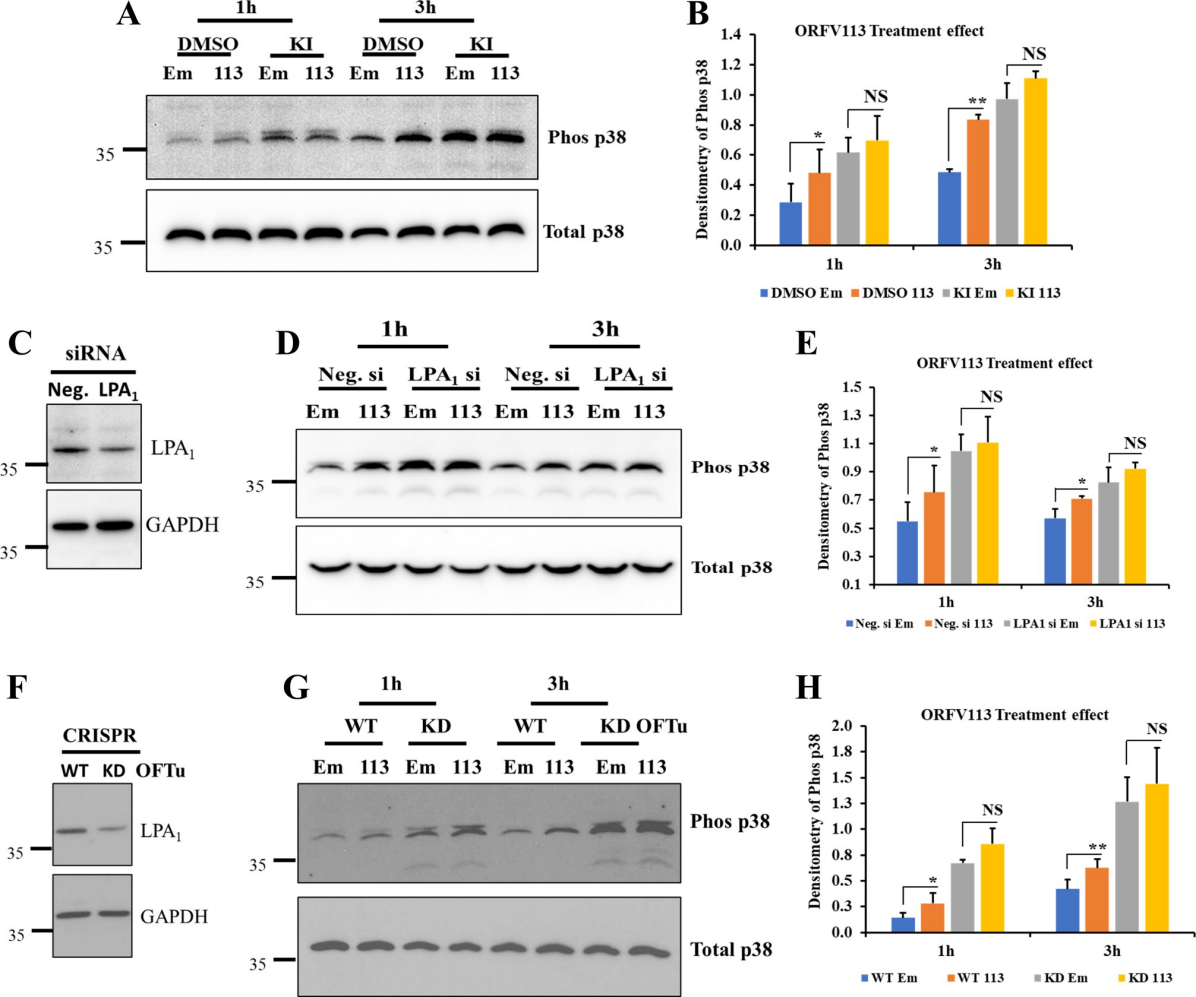
LPA_1_ is involved in ORV113 induced p38 phosphorylation. (A and B) Inhibiton of LPA_1_ blocks ORFV113 induced p38 activation. OFTu cells pretreated with DMSO (vehicle control) or KI16425 (LPA_1_ inhibitor) (10 μM) for 45 min were treated with immunoprecipitation products from pCMV-HA or pORFV113^HA^ expressing cells in presence of DMSO (vehicle control) or 10 μM KI16425 and harvested at 1 and 3 h post treatment. Total cell protein extracts were resolved by SDS-PAGE, blotted and incubated with antibodies against phos-p38 and total p38. (B) Densitometry of phos-p38 bands were normalized to total p38 (loading control). Results are average of three independent experiments. Error bars represent mean±SD. Statistical analysis was performed using paired Student’s t-test (*, *P<0*.*05*; **, *P<0*.*01*, *NS*: *non-significant*). (C, D and E) Effect of LPA_1_ receptor knockdown on ORFV113-induced p38 activation. OFTu cells transfected with universal negative control siRNA (Neg.) or with pooled LPA_1_ siRNA (100 nM) for 24 h were either harvested or treated with immunoprecipitation products from pCMV-HA or pORFV113^HA^ expressing cells and harvested at 1 h and 3 h. (C) To measure the LPA_1_ knockdown in siRNA-treated cells, total cell protein extracts were resolved by SDS-PAGE, blotted and incubated with antibodies against LPA_1_ and GAPDH. Densitometry of LPA_1_ bands were normalized to the loading control GAPDH. Approximately 45% LPA_1_ knockdown was routinely observed compared to universal negative control siRNA treatment. (D) To assess the effect of siRNA LPA_1_ knock down on ORFV113 induced p38 activation, total cell protein extracts were resolved by SDS-PAGE, blotted and incubated with antibodies against phos-p38 and total p38. (E) Densitometry of phos-p38 bands were normalized to the loading control total p38. Results are average of four independent experiments. Error bars represent mean±SD. Statistical analysis was performed as described above (*P < 0.*05*, *NS*: *non-significant*). (F, G and H) Effect of CRISPR LPA_1_ receptor knockdown on ORFV113 induced p38 activation. Wild type (WT) and CRISPR knock down (KD) OFTu cells cultured for 16 h were either harvested or treated with immunoprecipitation products from pCMV-HA or pORFV113^HA^ expressing cells and harvested at 1 h and 3 h. (F) LPA_1_ knockdown was measured as in C. Approximately 50% LPA_1_ knockdown was routinely observed. (G) The effect of LPA_1_ knockdown on ORFV113-p38 activation was assessed as in D. (H) Results are average of four independent experiments. Error bars represent mean±SD. Statistical analysis was performed as described above (*, *P<0*.*05*; **, *P<0*.*01*, *NS*: *non-significant*).

To examine the role of LPA_1_ on ORFV113 induced p38 activation, OFTu cells pretreated with DMSO (vehicle control) or LPA_1_ inhibitor KI16425 (10 μM) for 45 min were treated with immunoprecipitation products from pCMV-HA or pORFV113^HA^ expressing cells in presence of DMSO or KI16425 (10 μM), harvested at 1 h and 3 h post treatment and processed for Western blot. Significantly increased p38 phosphorylation was observed in vehicle control-treated cells in response to ORFV113 treatment compared to empty vector-treated cells, 1.7 fold at both 1 h and 3 h post treatment. However, no significant difference was observed between ORFV113 treatment compared to empty vector treated cells in presence of KI16425 (**Fig 7A and 7B**). Data indicate KI16425 inhibits ORFV113 induced p38 phosphorylation.

To assess the effect of LPA_1_ knockdown on ORFV113 induced p38 phosphorylation, OFTu cells were transfected with negative control siRNA or pooled LPA_1_ siRNA for 24 h. Cells were either harvested or treated with immunoprecipitation products from pCMV-HA or pORFV113^HA^ expressing cells, and p38 phosphorylation was assessed at 1 and 3 h post treatment by Western blot. Approximately 45% reduction in LPA_1_ routinely was observed in knock down cells (**Fig 7C**). Significantly increased p38 phosphorylation was observed in negative control siRNA transfected cells in response to ORFV113 treatment compared to empty vector treated cells (1.4 and 1.3-fold at 1 h and 3 h post treatment, respectively). However, no significant difference was observed between ORFV113 treatment compared to empty vector treated cells in LPA_1_ siRNA transfected cells (**Fig 7D and 7E**).

To further examine the effect of LPA_1_ knockdown on ORFV113 induced p38 phosphorylation, LPA_1_ knock down OFTu cells were obtained using CRISPR. Wild-type and CRISPR LPA_1_ knockdown OFTu cells were treated with immunoprecipitation products from pCMV-HA or pORFV113^HA^ expressing cells, harvested at 1 h and 3 h post treatment and assessed for p38 phosphorylation by Western blot. Approximately 50% reduction in LPA_1_ was routinely observed in knockdown cells (**Fig 7F**). Significantly increased p38 phosphorylation was observed in wild-type OFTu cells in response to ORFV113 treatment compared to empty vector treated cells (2 and 1.5-fold at 1 h and 3 h post treatment, respectively). However, no significant difference was observed between ORFV113 and empty vector treatment in LPA_1_ knockdown cells (**Fig 7G and 7H**). Together, these data suggest that LPA_1_ in cells inhibits p38 phosphorylation and binding of ORFV113 to LPA_1_ neutralizes its inhibitory function leading to increased p38 phosphorylation in cells mimicking that observed with KI16425 inhibition.

### Treatment with LPA and antibody against LPA_1_ enhances ORFV113 induced p38 phosphorylation in OFTu cells

**Fig 8:**
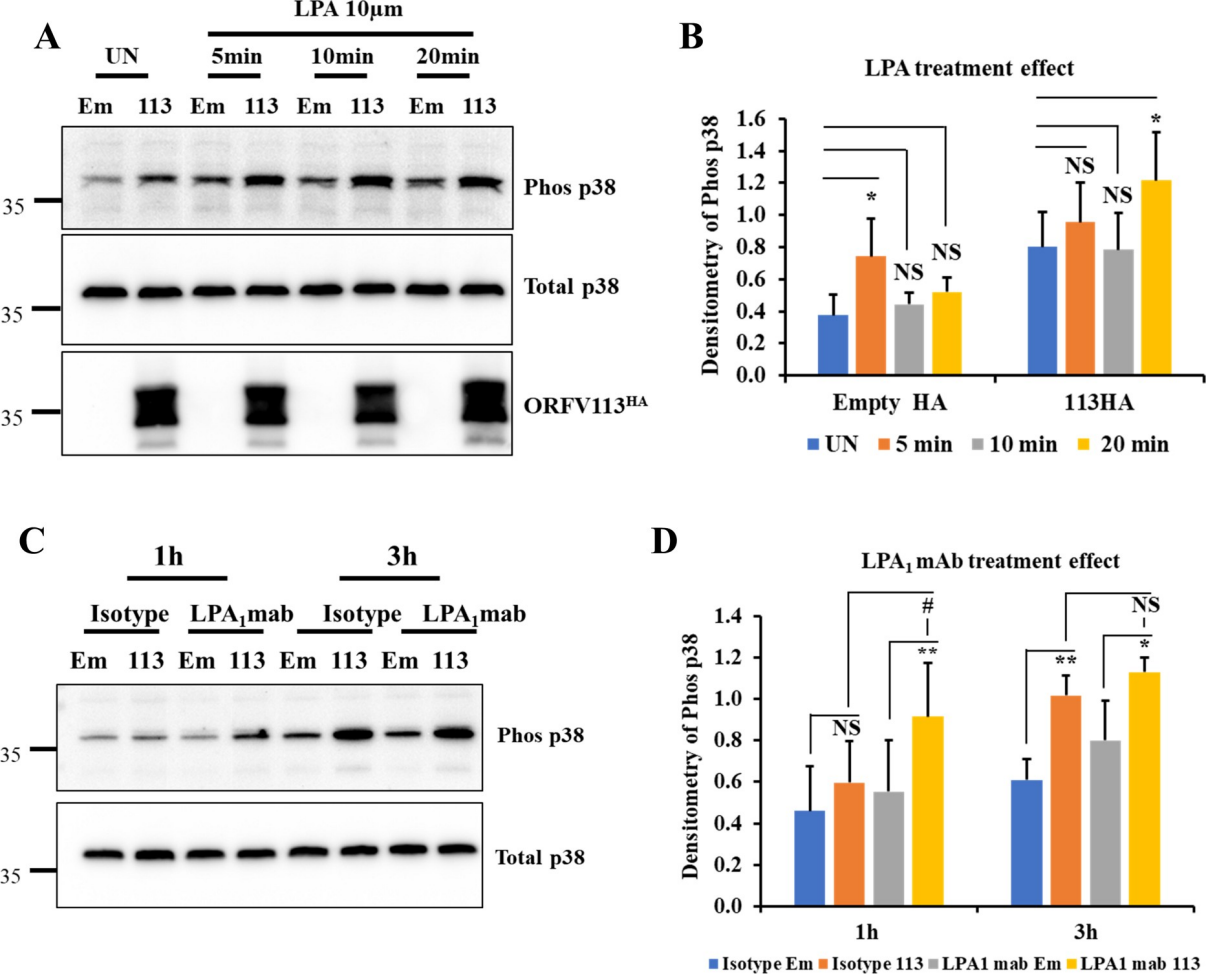
Treatment with LPA and antibody against LPA_1_ enhances ORFV113 induced p38 phosphorylation. (A and B) LPA treatment enhances ORFV113 induced p38 phosphorylation. OFTu cells transfected with plasmids pCMV-HA or pORFV113^HA^ for 5 h were induced with LPA harvested at 5, 10 and 20 min. Total cell protein extracts were resolved by SDS-PAGE, blotted and incubated with antibodies against phos-p38, total p38, HA and GAPDH. (B) Densitometry of phos-p38 bands were normalized to total p38 (loading control). Results are average of four independent experiments. Error bars represent mean±SD. Statistical analysis was performed using one-way ANOVA with post hoc Tukey test for multiple comparisons (*, *P<0*.*05*; **, *P<0*.*01*, *NS*: *non-significant*). (C and D) Antibody against LPA_1_ enhances ORFV113 induced p38 phosphorylation. OFTu cells pretreated with isotype control or LPA_1_ monoclonal antibody (10 μg/ml) for 45 min were treated with immunoprecipitation products from pCMV-HA or pORFV113^HA^ expressing cells in presence of isotype control or LPA_1_ monoclonal antibody (10 μg/ml) and harvested at 1 and 3 h. Total cell protein extracts were resolved by SDS-PAGE, blotted and incubated with antibodies against phos-p38 and total p38. (D) Densitometry of phos-p38 bands were normalized as in B. Results are average of five independent experiments. Error bars represent mean ±SD. Statistical analysis was performed as described in B (*,^#^, *P<0*.*05*; **, *P<0*.*01*; *NS*: *non-significant*).

Results above indicate that LPA_1_ is involved in ORFV113 induced p38 phosphorylation. Several studies have shown that LPA induces p38 phosphorylation in different cell types [32–35]. In the skin, LPA levels increase in response to tissue injury and induces multiple signaling pathways including the p38 pathway [50]. Thus, a potential role of LPA in ORFV113-induced p38 signaling in natural infection can be hypothesized. To examine the effect of LPA on ORFV113-induced p38 signaling, OFTu cells transfected with plasmids pCMV-HA or pCMV-ORFV113^HA^ for 5 h were treated with LPA for 5, 10 or 20 min, harvested and processed for western blot. In cells transfected with control plasmids, significantly increased p38 phosphorylation was observed in cells treated with LPA for 5 min (1.8-fold) compared to untreated cells. Significantly increased p38 phosphorylation was observed in ORFV113 expressing cells treated with LPA for 20 min (1.6 -fold) compared to untreated cells (**Fig 8A and 8B**). Data indicate that LPA enhances ORFV113-induced p38 signaling.

To assess the involvement of LPA_1_ on ORFV113 induced p38 phosphorylation, OFTu cells pretreated with isotype control or LPA_1_ monoclonal antibody for 45 min were treated with immunoprecipitation products from pCMV-HA or pORFV113^HA^ expressing cells in presence of antibodies and harvested at 1 h and 3 h, and processed for Western blot. Treatment with antibody against LPA_1_ and with ORFV113 significantly increased p38 phosphorylation compared to isotype control/ORFV113 treated cells at 1 h post treatment (1.5-fold) (**Fig 8C and 8D**). Data suggests that treatment with antibody against LPA_1_ enhances ORFV113 induced p38 phosphorylation.

### ORFV113 is a major virulence determinant in the natural host

**Fig 9:**
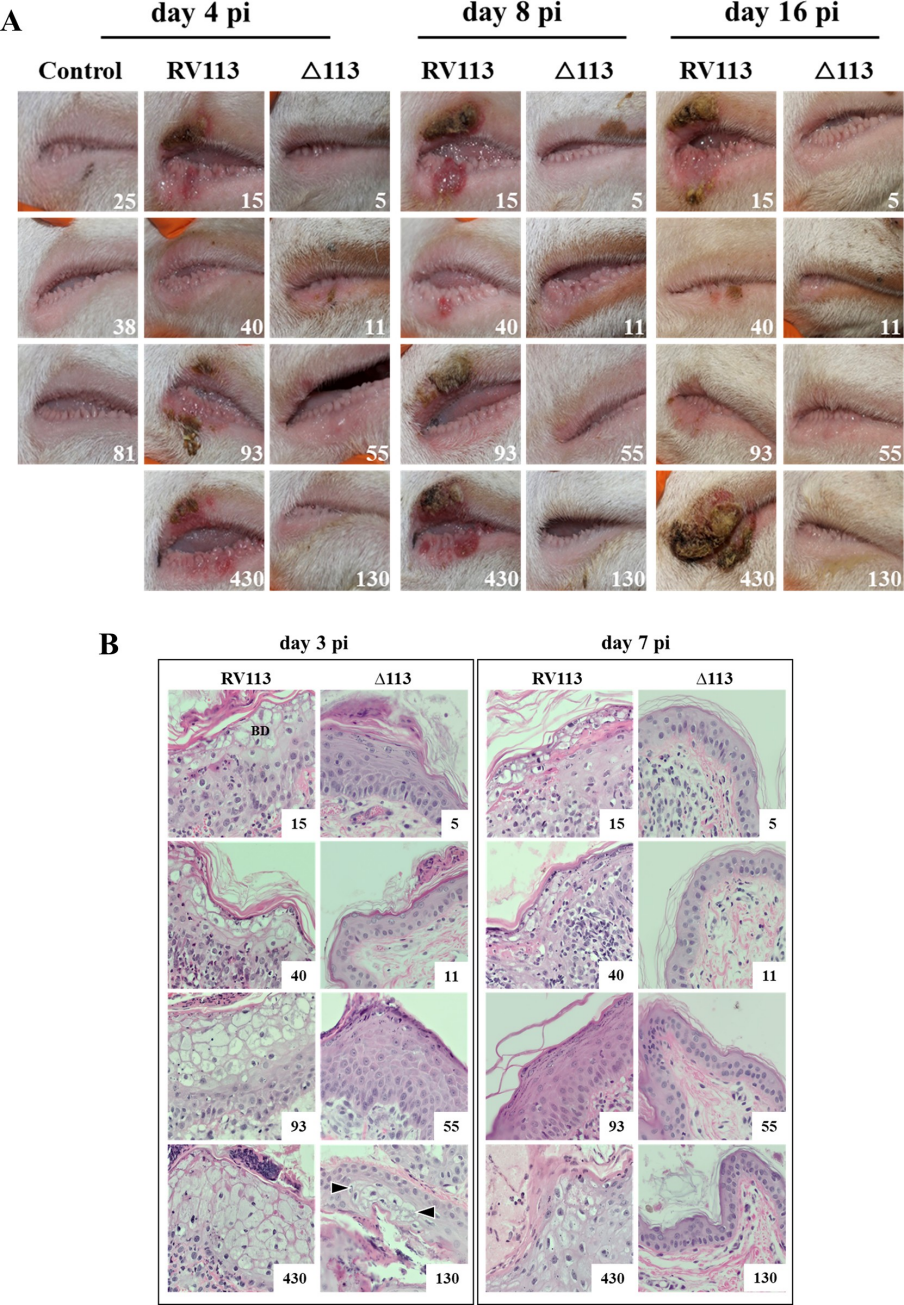
Effect of ORFV113 on virus virulence and pathogenesis in sheep. (A) Sheep were scarified in the right labial commissure, inoculated with virus OV-IA82RV113^HA^ (RV113; sheep # 15, 40, 93, and 430) or OV-IA82Δ113 (Δ113; sheep # 5, 11, 55 and 130), or treated with PBS (control; sheep # 25, 38 and 81), and monitored for 20 days. Gross changes are shown for days 4, 8 and 16 p.i. (B) Histological changes in the skin of the inner side of thighs following inoculation with OV-IA82RV113^HA^ (RV113) or OV-IA82Δ113 (Δ113). Representative histopathological changes are shown for days 3 and 7 p.i. H&E staining, X 400. BD, area of ballooning degeneration; arrowheads, restricted BD.

The effect of ORFV113 in disease outcome was investigated in sheep, a natural ORFV host. Groups of animals were inoculated with deletion mutant virus OV-IA82Δ113 (n = 4), revertant virus OV-IA82RV113^HA^ (n = 4) or PBS (control group, n = 3) in the right labial commissure and the inner side of the thighs, and disease course was monitored for 20 days. Sheep in the revertant virus group exhibited gross changes in the lip compatible with orf as early as day 2 p.i., with mild erythema (#40), or erythema, papulae, and pustules (#15, #93 and # 430) at inoculation sites. By day 4 p.i., lesions exacerbated in all sheep in the group except sheep #40, in which erythema regressed (**Fig 9A**, RV113). Lesions in sheep #15, #93 and # 430 continued to expand and by day 8 p.i. showed marked scabby tissue deposition in the upper lip, while sheep #40 showed a single small papule in the lower lip. On day 16 p.i., lesions in sheep #40 and #93 regressed, while those in sheep #15 and #430 continued to enlarge by further scabby tissue deposition (**Fig 9A**). By day 20 p.i. (end point of the experiment), lesions in sheep #15 and #430 abated. Similar to control sheep, and in marked contrast with revertant virus-infected sheep, the four animals infected with OV-IA82Δ113 did not exhibit any sign of ORFV infection during the course of the experiment (**Fig 9A**, Δ113). This indicates that ORFV113 is a significant ORFV virulence determinant.

**Fig 10:**
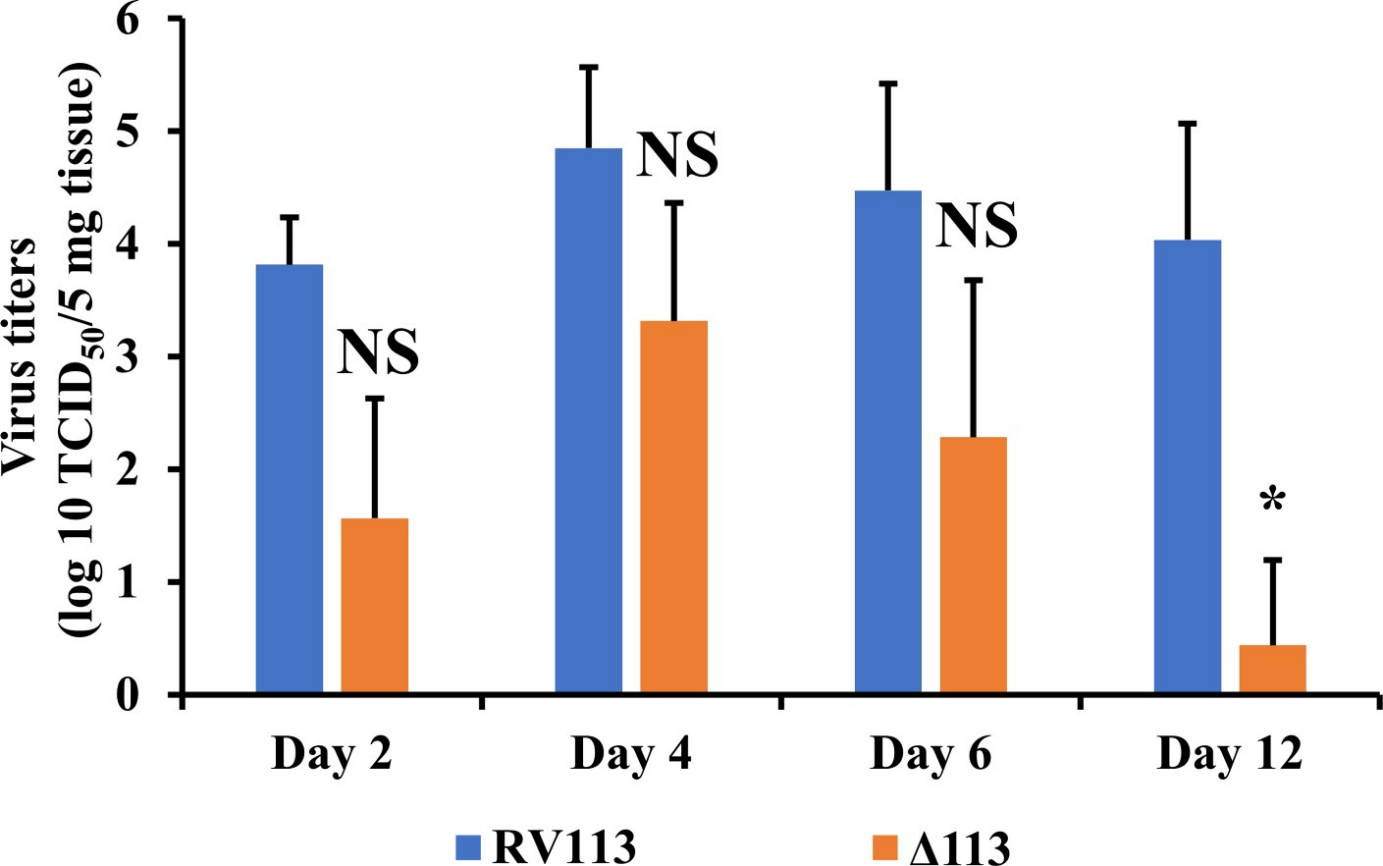
Effect of ORFV113 on viral titers of ORFV-infected animals. Skin biopsies were collected from animals infected with viruses OV-IA82Δ113^GFP^ (Δ113) or OV-IA82RV113^HA^ (RV113), or mock infected at various times p.i. Titers were determined as described in material and methods, and expressed as TCID_50_/5mg tissue. Error bars represent mean±SD. Statistical analysis was performed using paired Student’s t-test (*, *P<0*.*05*, *NS*: *non-significant*).

Punch biopsies were collected from inoculation sites in the thighs at 1, 2, 3 and 7 days p.i. and processed for histology. By day 2 p.i., samples from all animals showed changes associated with skin scarification, including epidermal hyperplasia, active re-epithelialization, and various degrees of neutrophil infiltration. Zero of three, 3 of four, and 2 of four sheep in control, revertant virus, and deletion mutant virus groups, respectively, exhibited small foci of keratinocytes with ballooning degeneration (BD) in the stratum spinosum of the epidermis, which represent dying cells containing progeny virions as previously described [51]. By day 3 p.i., multiple expanding BD sites were observed in all four sheep infected with revertant virus (**Fig 9B**, left panel, RV113). In contrast, BD sites in the deletion mutant group were not seen in two sheep (#11 and 55), and remained restricted in size in sheep #5 and #130 (as an example for restricted BD, see arrowheads in sheep #130). In addition, skin infiltrates of inflammatory cells were more populated in the revertant group than in the deletion mutant group (**Fig 9B**, compare the two columns in left panel). By day 7 p.i., three of the sheep in the revertant virus group (# 15, # 40, and # 430) and none of the sheep in the deletion mutant virus group exhibited epidermal areas of BD and significant inflammatory cell infiltration (**Fig 9B**, right panel). No ORFV-associated changes were observed in control group samples during the experimental time. Together, data indicate that infection of sheep with OV-IA82Δ113, a virus lacking ORFV113, is controlled by the host before gross lesions and significant histopathological changes take place. Consistent with this, OV-IA82Δ113 titers in skin biopsy samples were approximately, 10–100 fold lower than revertant virus titers in the first week post infection, and 1,000–10,000 fold lower by day 12 p.i. (**Fig 10**).

### ORFV113 enhances p38 signaling in ORFV-infected keratinocytes *in vivo*

**Fig 11:**
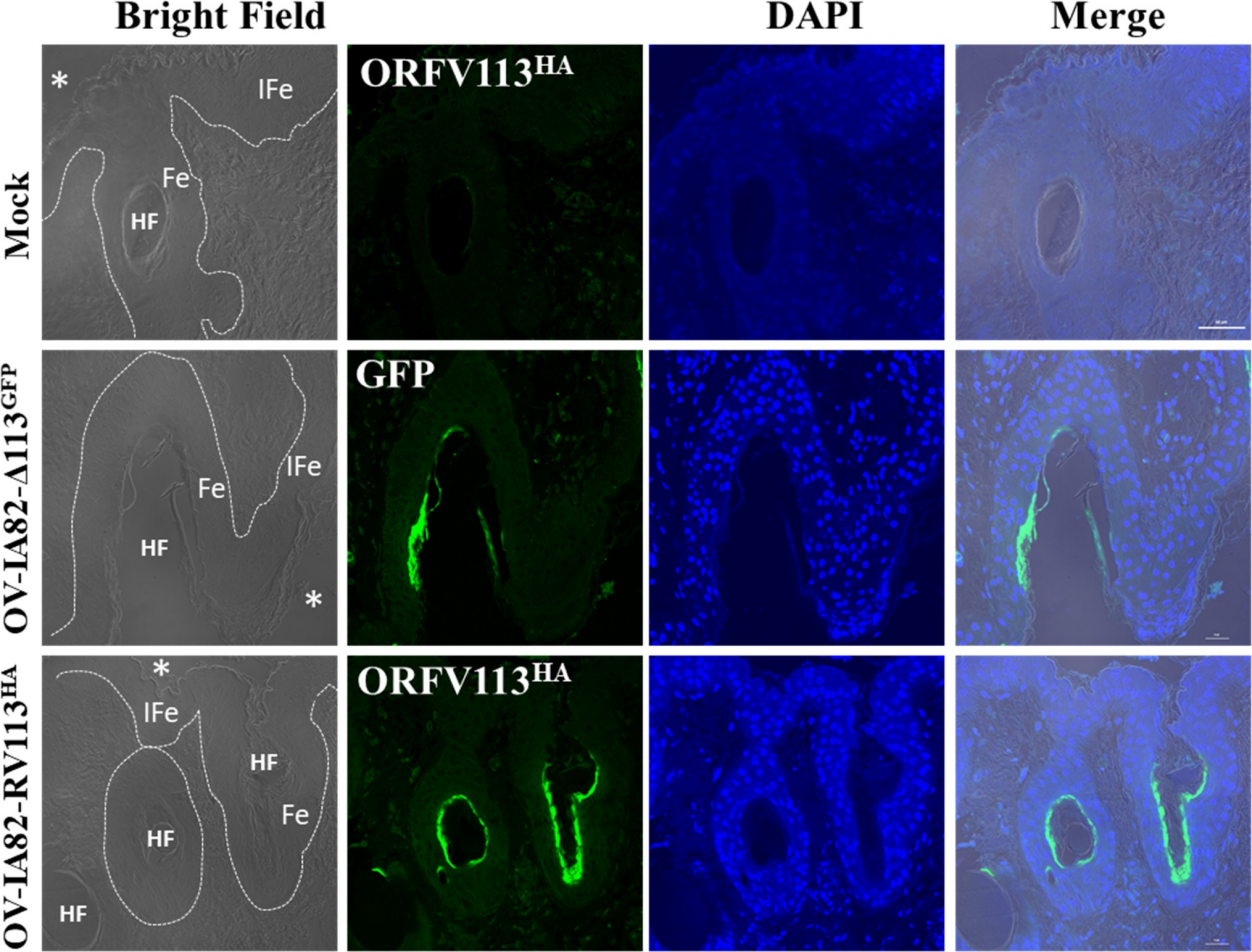
ORFV infects the infundibular region of hair follicles at early times p.i. Sheep were scarified in the inner side of the thighs and infected with viruses OV-IA82Δ113^GFP^ or OV-IA82RV113^HA^, or mock infected, and tissue samples collected at various times post-infection. Shown are representative confocal microscopy images at 24 h post-infection. *, outer side of the skin; dashed line, location of the basal membrane of epithelium; HF, lumen of hair follicles at the level of infundibular region; IFe, interfollicular epidermis; Fe, follicular epidermis; Green: GFP or ORFV113^HA^, Blue: DAPI. Magnification 40X.

**Fig 12:**
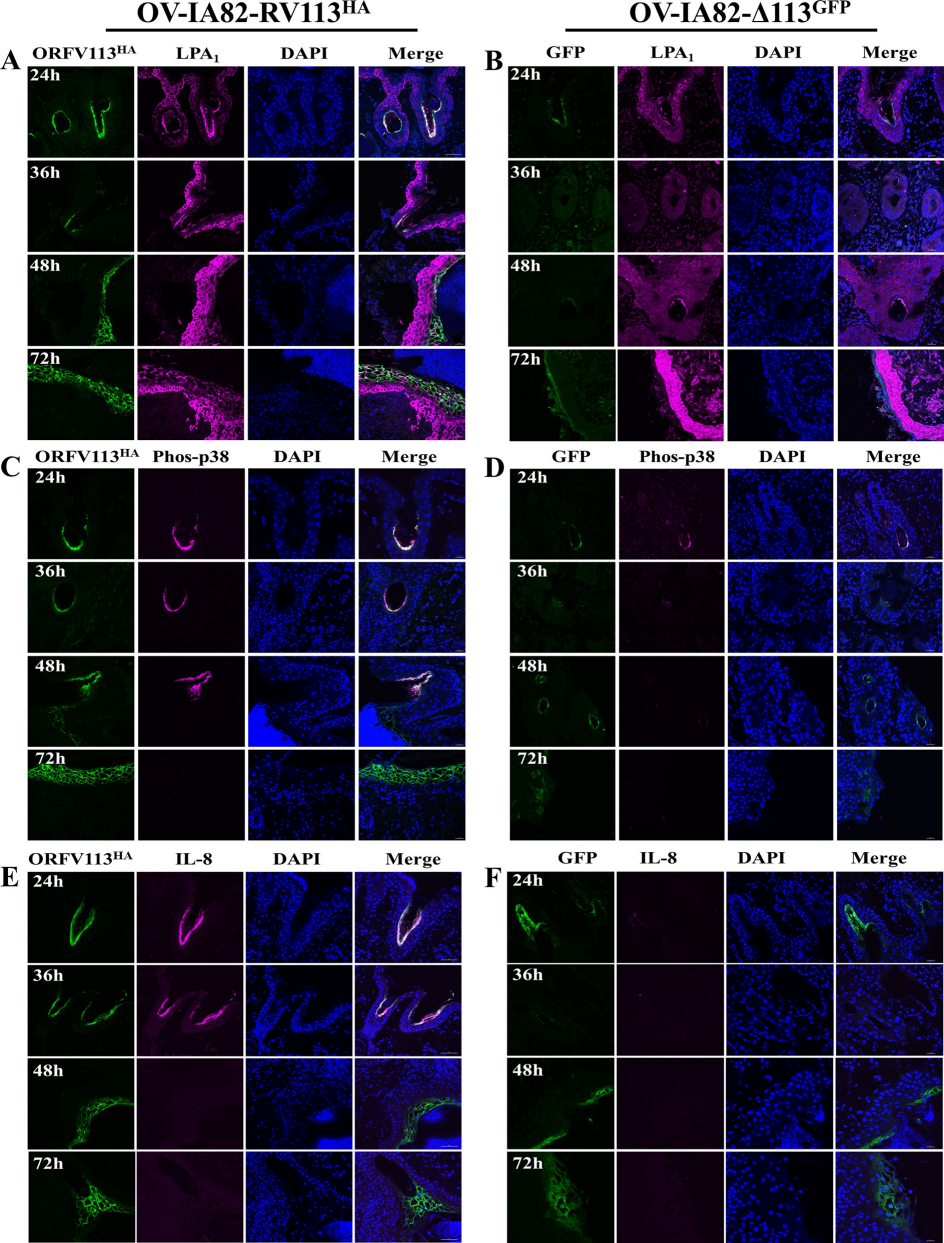
Effect of ORFV113 on p38 signaling in the skin of ORFV-infected animals. Immunofluorescence was performed on skin sections of animals infected with OV-IA82RV113^HA^ (RV113) or OV-IA82Δ113^GFP^ (Δ113), or mock infected at various times p.i as described in materials and methods. (A and B) LPA_1_ Expression (A) RV113 and Δ113 (B), Green: ORFV113^HA^ or GFP, Violet: LPA_1_, Blue: DAPI; (C and D) Level of phos-p38 (C) RV113 and Δ113 (D), Green: ORFV113^HA^ or GFP, Violet: phos-p38, Blue: DAPI.; (E and F) IL-8 expression (E) RV113 and Δ113 (F), Green: ORFV113^HA^ or GFP, Violet: IL-8, Blue: DAPI.

To investigate the effect of ORFV113 on p38 signaling in the skin, skin sections from ORFV-infected animals were examined by immunofluorescence and confocal microscopy. We first determined that cells infected with OV-IA82RV113^HA^ or OV-IA82Δ113 (a virus expressing GFP) can be detected in the most apical layer of the hair follicle infundibulum as early as 24 h p.i (**Fig 11**). Next, expression of LPA_1_, phos-p38 and IL-8 was examined on skin sections from control and infected animals at 24, 36, 48 and 72 h. Increased LPA_1_ expression was observed in keratinocytes infected with OV-IA82RV113^HA^ at 24, 36 and 48 h p.i., suggesting that ORFV113 induces expression of LPA_1_ (**Fig 12A and 12B**). Likewise, keratinocytes infected with OV-IA82RV113^HA^ but not with OV-IA82Δ113 showed increased levels of phos-p38 and IL-8 compared to non-infected cells, indicating that ORFV113 induces p38 phosphorylation and IL-8 expression (**Fig 12C–12F**). Together, data suggest that ORV113 enhances p38 signaling in the epidermis of ORFV infected animals.

## Discussion

LPA signaling in the skin is known to affect a wide range of cellular functions, including cell differentiation, migration, proliferation and chemotaxis, and it has been implicated in innate immune responses, inflammation and wound healing [21–25]. Here, we describe a novel ORFV protein, ORFV113, which interacts with the GPCR LPA_1_ receptor (**Fig 3**) and enhances kinase p38 phosphorylation and signaling in ORFV-infected cells, cells transiently expressing ORFV113 and cells treated with soluble ORFV113 (**Figs 4 and 6**).

Some viruses have evolved mechanisms to hijack host GPCR signaling. Several herpes viruses, including Kaposi’s sarcoma-associated herpesvirus (KSHV) [52], human cytomegalovirus (HCMV) [53], and Epstein–Barr virus (EBV) [54], encode constitutively active GPCRs homologs (vGPCR) that allow viruses to activate multiple cellular signaling pathways akin to host GPCR. Other viruses use GPCRs as co-receptors for cell entry. For example, GPCRs CXCR4 and CCR5 are essential for HIV fusion and entry into target cells [55].

Viral modulators of p38 signaling have been described but none have been linked to LPA-p38 signaling pathway. For example vaccinia virus protein A52R, was found to activate p38 MAPK in a TRAF6 dependent manner and vaccinia virus E3 suppressed p38 phosphorylation in infected cells via an unknown mechanism [39–41]. Additional viral proteins modulating p38 signaling have been described. Chikungunya virus protein nsP2 interacted with phos-p38 and phos-JNK protein [56]. Herpes simplex virus type-1 (HSV-1) virion transactivator protein VP16 stimulated p38/JNK activation, however the mechanism of action is not known [57]. Expression of HSV-1 protein ICP27 alone directly activated p38 signaling, leading to stimulation of the host cell apoptotic pathways [58]. Signaling by KSHV-encoded GPCR resulted in p38 activation, leading to phosphorylation of hypoxia-inducible factor 1 alpha and resulting in increased expression of vascular endothelial growth factor [59].

We observed significantly decreased levels of p38 phosphorylation when cells were infected with OV-IA82Δ113 suggesting that ORFV113 enhances p38 signaling in infected cells (**Fig 4**). Consistent with this observation, decreased levels of phos-p38 and IL-8 at early times p.i. were observed in animals infected with OV-IA82Δ113 (**Fig 12C–12F**). These data indicate that ORFV113 expression enhances phosphorylation of p38 and subsequent signaling events (i.e increased IL-8 transcription) both *in vitro* and *in vivo (***Figs 6C, 6D, 12E and 12F**).

**Fig 13:**
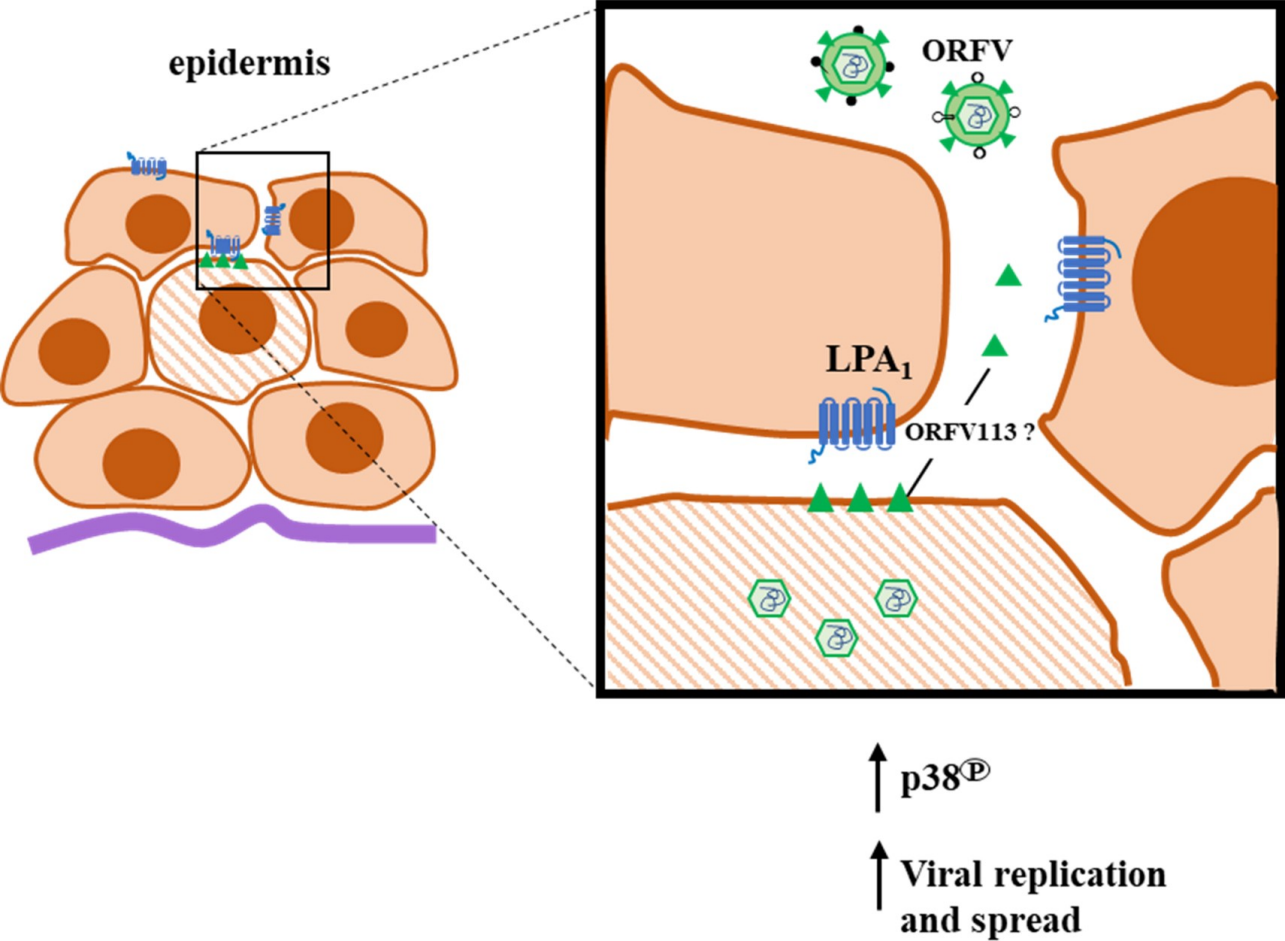
Proposed function of ORFV113 during infection of the epidermis by ORFV. LPA-p38 signaling targeted by ORFV113 might exert an effect on p38 signaling in neighboring cells in a virus plaque or focal lesion in ORFV infected epidermis. ORFV113 interacts with GPCR, LPA_1_ and enhances p38 phosphorylation in ORFV infected cells. ORFV113 expressed in the membrane of infected cells might be interacting with LPA_1_ on adjacent cells in a juxtacrine manner, leading to p38 phosphorylation in neighboring cells. Alternatively, soluble ORFV113 may perform the same function in a paracrine manner as ORFV113 is present in the supernatant of infected cells. The mechanism by which ORFV113 is present in the supernatant is yet to be determined.

Data show that ORFV113 is nonessential for ORFV replication in OFTu cells *in vitro* (**Fig 2A and 2B**) but is important for virus replication in keratinocytes *in vivo* (**Fig 10**). Notably, ORFV113 is important for ORFV plaque formation in cultured cells (**Fig 2C and 2D**), suggesting that expression of ORFV113 in infected cells and possibly signaling induced by ORFV113 in neighboring cells are important during ORFV cell-to-cell spread (**Fig 13**). It is tempting to speculate that the restricted size and small number of infection foci seen at 2 and 3 days p.i. in sheep infected with OV-IA82Δ113 (**Fig 9B**) reflect this same limited ability of virus to spread.

Consistent with the observation above, plaque assay and virus growth data in presence of a p38 kinase inhibitor (**Fig 5**) indicate that p38 signaling is essential for ORFV replication and normal plaque formation. As expected, the effect of the p38 inhibitor was not as pronounced on plaque size and viral yields of OV-IA82Δ113, a virus deletion mutant lacking ORFV113, compared to the wildtype virus, suggesting that ORFV113 enhances ORFV susceptibility to the effect of p38 inhibitor and may be involved in regulating p38 signaling during ORFV infection.

It is generally accepted that ORFV infection requires a severed skin or mucosa that expose activated keratinocytes to inoculum virus [2,60,61]. Prickly plants or stubble contaminated with shed scabs, and animals with active lesions are the usual sources of virus. The notion of epidermal damage as a prerequisite for infection is reinforced by studies showing that typical orf lesions developed after hair plucking or skin scarification followed by topical virus inoculation, but not after intradermal, subcutaneous or intravenous virus injection, or topical virus inoculation of intact skin [2]. The strategies of ORFV to ensure proper virus replication in the context of active host inflammatory and repair responses at virus entry sites are unknown. LPA levels significantly increase in areas with skin damage [50] and animal studies have shown that LPA reduces the duration of skin wound healing [62–64]. LPA has been shown to induce the migration, proliferation and differentiation of keratinocytes and dermal fibroblasts, which are essential processes underlying skin tissue repair and reconstruction [65–67]. We can speculate an interplay between ORFV113 and LPA_1_ affecting downstream events of LPA signaling.

Infection of sheep with deletion mutant virus OV-IA82Δ113 resulted in striking disease attenuation compared with wildtype virus infection (**Fig 9**). This was reflected by the absence of gross lesions, the temporally and spatially restricted histopathological changes, and the markedly reduced viral load, most likely a consequence of the poor OV-IA82Δ113 replication, spread, or both (**Figs 9, 10 and 12**). Further studies will be required to investigate the interplay of LPA signaling and ORFV113 in the context of ORFV pathogenesis *in vivo*.

Previously, ORFV antigen has not been detected *in vivo* before 2 days post-infection using the scarification model [68]. Here, we detected viral antigen at 24 h p.i., with signal consistently associated with the apical layer of the outer root sheath (ORS) in the infundibular region of hair follicles (HF) (**Figs 11 and 12**), suggesting that keratinocytes in this layer are more sensitive to virus infection than keratinocytes in the interfollicular (superficial) epidermis (IFE). This was unexpected since ORFV is thought to target activated keratinocytes associated with repair responses in the IFE and HF’s ORS [2,69]. In contrast, the apical layer of the HF infundibulum is thought to be fully differentiated and as such it represents a very different cell environment for virus replication. The infundibular region of HFs is a major site of chemokine and antimicrobial peptide production, and expresses high levels of toll-like receptors [70]. Our *in vivo* data showed transient p38 activation early in infection (**Fig 12C and 12D**), suggesting that this may be a requirement for early viral replication in these differentiated infundibular epithelial cells. Further studies are required to understand early events of ORFV infection *in vivo*.

The fact that ORFV113 is a virion component suggests it performs an important function(s) in infected cells, which likely involves early targeting of LPA_1_ and/or other cellular proteins (**Fig 1B**). ORFV113 expressed in the membrane of infected cells might be interacting with LPA_1_ on adjacent cells in a juxtacrine manner, leading to p38 phosphorylation in neighboring cells (**Figs 1E, 3B and 3C**). Alternatively, soluble ORFV113 may perform the same function in a paracrine manner as ORFV113 is present in the supernatant of infected cells (**Fig 1C**). The mechanism by which ORFV113 is present in the supernatant is yet to be determined. Thus, LPA-p38 signaling targeted by ORFV113 affects ORFV replication and spread both *in vitro* and *in vivo* (**Fig 13**).

## Supporting information

S1 Fig Clustal W alignment of PPV113 amino acid sequences. Aligned sequences are ORFV strains OV-IA82, NZ2, OV-SJ1, OV-XY, OV-SA00, OV-GO, D1701, NA1/11, B029, NP, NA17, and SY17, PCPV strains F00.120R, It1303/05, and VR634, BPSV strains BV-AR02, BV-TX09c5 and BV-TX09c15 and red deerpox virus strain HL953 and sealpox virus strain AFK76s1 (see Materials and Methods for accession numbers). The boxed sequences correspond to predicted transmembrane domains. Asterisks [*], colons [:], and periods [.] below the alignment indicate fully, strongly, and weakly conserved, residues, respectively.
(TIF)

S2 Fig ORFV113 expression in reducing and non-reducing conditions. OFTu cells mock infected or infected with revertant virus OV-IA82-RV113^Flag^ (MOI, 10) were harvested at 24 h p.i. Total cell protein extracts were resolved by SDS-PAGE in reducing and non-reducing conditions, blotted and incubated with antibody against Flag. Result is representative of two independent experiments.
(TIF)

S3 Fig Transcriptional kinetics of ORFV113. Transcription kinetics of ORFV113, ORFV055 (late gene control) and ORFV119 (early gene control) was assessed during ORFV infection in OFTu cells in the presence (+) or absence (-) of AraC. Cells infected with OV-IA82 (MOI = 10) were harvested at respective times and transcription levels were determined by RT-PCR. Results is representative of two independent experiments.
(TIF)

S4 Fig Replication characteristics of ORFV113-gene deletion virus at MOI 0.01. OFTu cells were infected with wildtype OV-IA82 (WT), ORFV113 deletion mutant OV-IA82Δ113 (Δ113) or revertant OV-IA82RV113^Flag^ (RV113) viruses at MOI 0.01. Titers were determined at indicated times p.i. and expressed as TCID_50_/ml. Result is representative of two independent experiments.
(TIF)

S5 Fig Cellular interactors of ORFV113. OFTu cells transfected with plasmid pCMV-ORFV113^Flag^ was harvested at 24 h post transfection and total cell protein was extracted. Immunoprecipitation was performed using anti-flag antibody. LC-MS Mass Spectrometry was performed, and data were analysed using in house Mascot server. Cut off score was 3 at p<0.05. emPAI (Relative abundance)
(TIF)

S6 Fig Co-immunoprecipitation of CD2v with LPA_1_. OFTu cells co-transfected with plasmids pcDNA3.1^His^ (pHis)and pCMV-HA (pHA) (Empty plasmids) or pcDNA3.1-LPA_1_^His^ (pLPA_1_^His^) and pCMV-CD2v^HA^ (pCD2v^HA^) were harvested at 24 h post transfection and membrane fractions were extracted. Membrane lysates (left) and extracts immunoprecipitated with anti-His (upper panel) or anti-HA (lower panel) antibodies were analyzed by SDS-PAGE-Western blotting with antibodies directed against proteins indicated on the right. * and ** denote light and heavy chain of the IgG antibody, respectively.
(TIF)

S7 Fig Effect of ORFV113 on p38 phosphorylation in infected cells at 12 and 24 h p.i. (A) OFTu cells were mock-infected or infected with OV-IA82 (WT), OV-IA82-Δ113 (Δ113), OV-IA82-RV113^HA^ (HA) and OV-IA82-RV113 (UN) (MOI:10) for 12 and 24 h p.i. Total cell protein extracts were resolved by SDS-PAGE, blotted and incubated with antibodies against phos-p38 and total p38. (B) Densitometric analysis of bands corresponding to phosphorylated p38. Densitometry of phos-p38 bands were normalized to the loading control total p38. Results are the average of three independent experiments. Error bars represent mean±SD. Statistical analysis was performed using one-way ANOVA with post hoc Tukey test for multiple comparisons (*, *P<0*.*05*; **, *P<0*.*01*, *NS*: *non-significant*). * denote statistical significance compared to mock. Statistical comparison of a treatment to WT is shown with a line drawn over the respective treatment bar.
(TIF)

S8 Fig Supernatant from ORFV113 expressing cells induces p38 phosphorylation. (A) Culture supernatants were harvested from OFTu cells transfected with pCMV-HA (Empty HA plasmid), pCMV-ORFV113^HA^ (ORFV113^HA^ plasmid) or pCMV-ORFV113 (Untagged ORFV113 plasmid) (UN113) at 24 h post transfection. Naive OFTu cells were then treated with respective clarified supernatants and harvested at 3 h post treatment. Total cell protein extracts were resolved by SDS-PAGE, blotted and incubated with antibodies against phos-p38 and total p38. (B) Densitometric analysis of bands corresponding to phosphorylated p38. Densitometry of phos-p38 bands were normalized to total p38. Results are the average of three independent experiments. Error bars represent mean±SD. Statistical analysis was performed using one-way ANOVA with post hoc Tukey test for multiple comparisons (*, *P<0*.*05*; **, *P<0*.*01*).
(TIF)
