# Supplementary figures and images for: The novel ORFV protein ORFV113 activates LPA-p38 signaling
Source: PLoS Pathog. 2021 Oct 6;17(10):e1009971. doi: 10.1371/journal.ppat.1009971 (PMC8523077; doi:10.1371/journal.ppat.1009971)

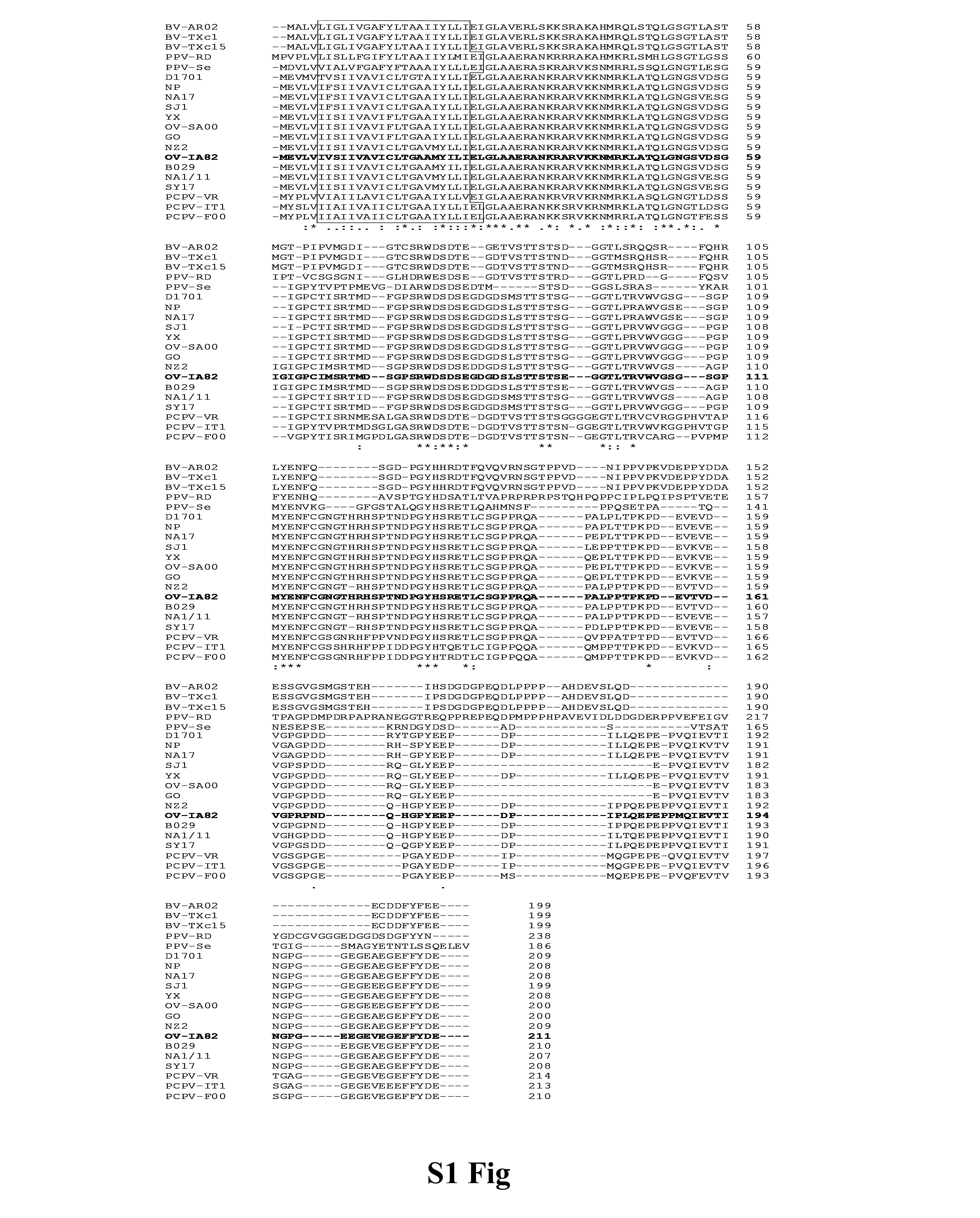

Supplement: S1 Fig — Aligned sequences are ORFV strains OV-IA82, NZ2, OV-SJ1, OV-XY, OV-SA00, OV-GO, D1701, NA1/11, B029, NP, NA17, and SY17, PCPV strains F00.120R, It1303/05, and VR634, BPSV strains BV-AR02, BV-TX09c5 and BV-TX09c15 and red deerpox virus strain HL953 and sealpox virus strain AFK76s1 (see Materials and Methods for accession numbers). The boxed sequences correspond to predicted transmembrane domains. Asterisks [*], colons [:], and periods [.] below the alignment indicate fully, strongly, and weakly conserved, residues, respectively. (TIF) [file ppat.1009971.s001.TIF]

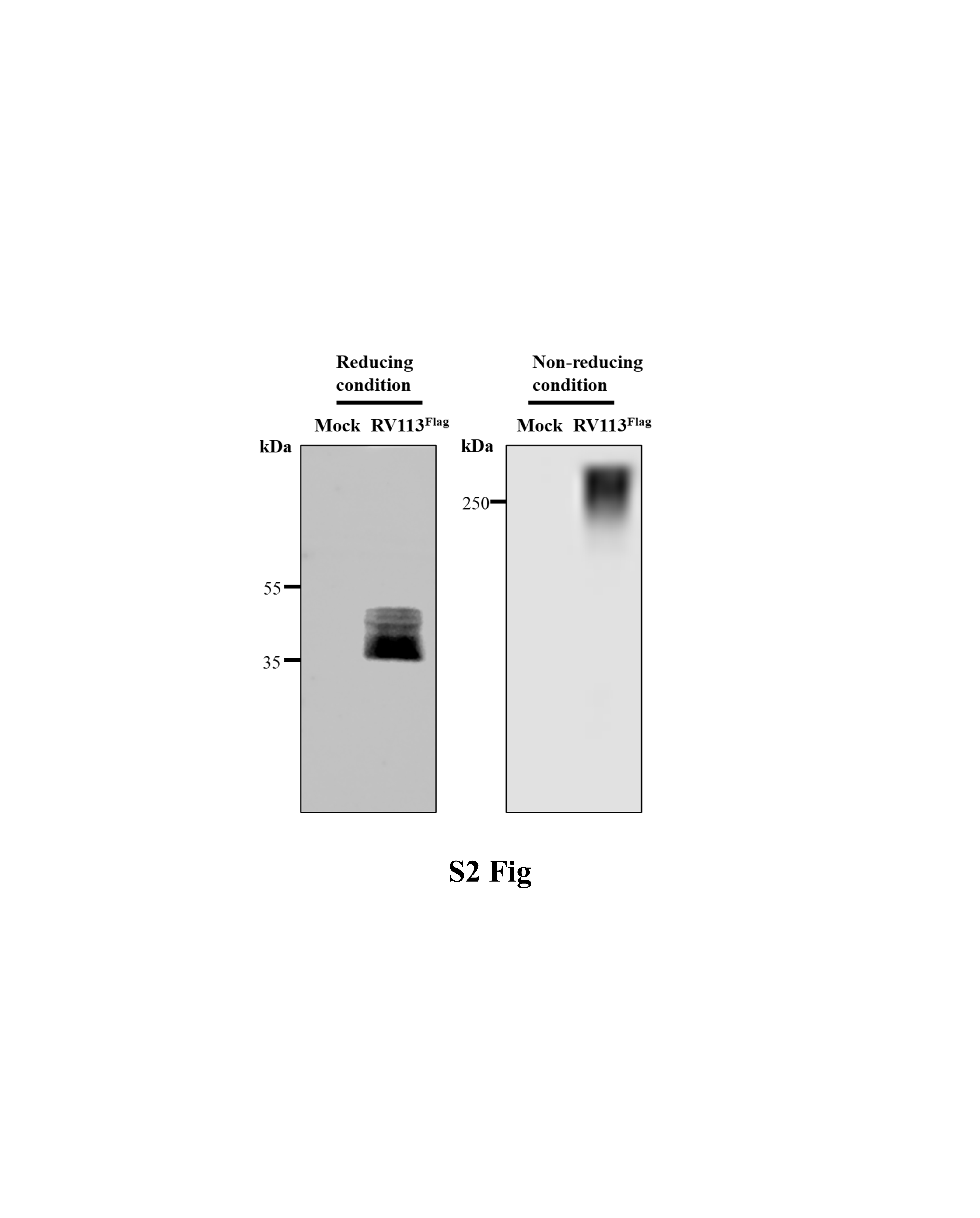

Supplement: S2 Fig — OFTu cells mock infected or infected with revertant virus OV-IA82-RV113Flag (MOI, 10) were harvested at 24 h p.i. Total cell protein extracts were resolved by SDS-PAGE in reducing and non-reducing conditions, blotted and incubated with antibody against Flag. Result is representative of two independent experiments. (TIF) [file ppat.1009971.s002.TIF]

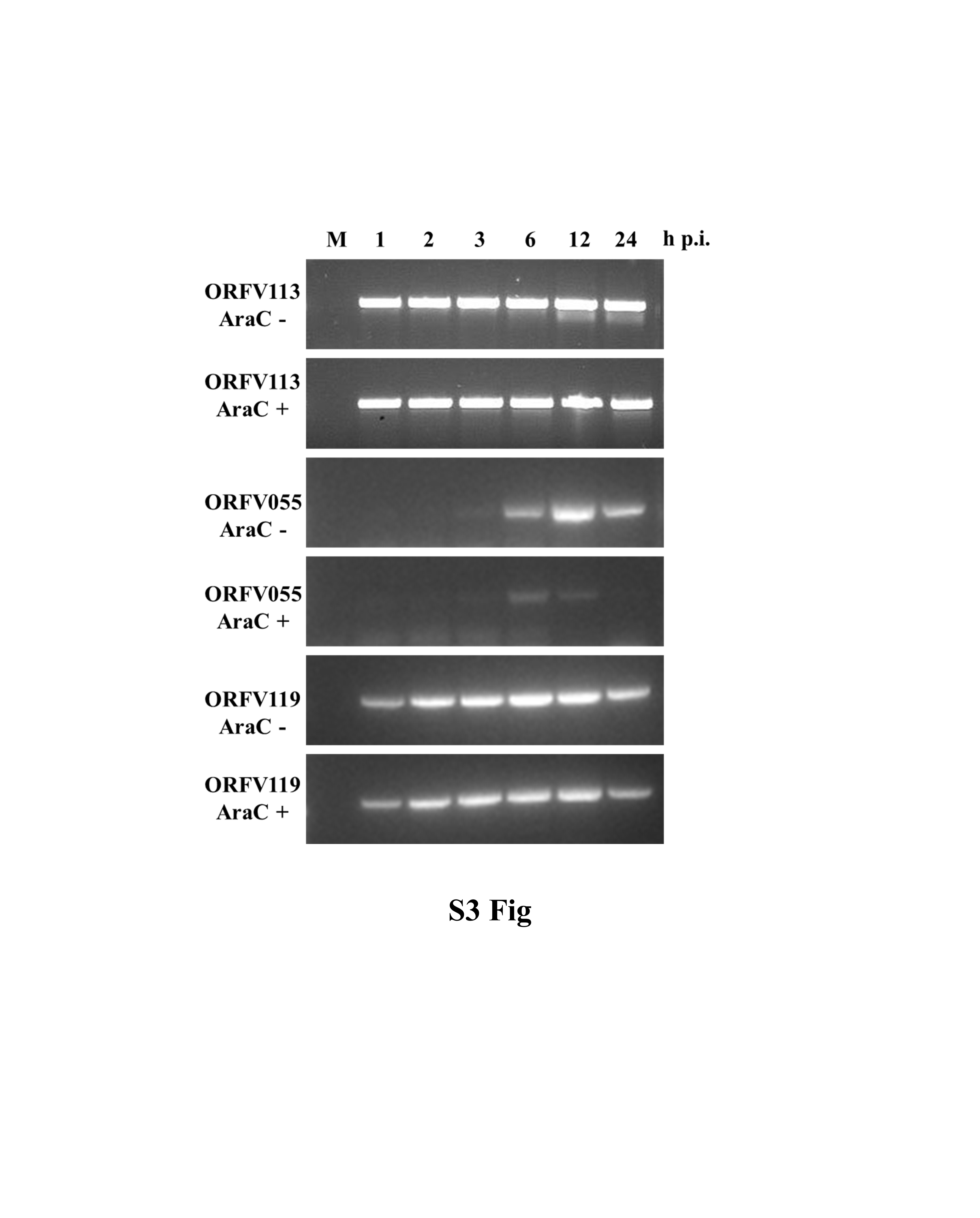

Supplement: S3 Fig — Transcription kinetics of ORFV113, ORFV055 (late gene control) and ORFV119 (early gene control) was assessed during ORFV infection in OFTu cells in the presence (+) or absence (-) of AraC. Cells infected with OV-IA82 (MOI = 10) were harvested at respective times and transcription levels were determined by RT-PCR. Results is representative of two independent experiments. (TIF) [file ppat.1009971.s003.TIF]

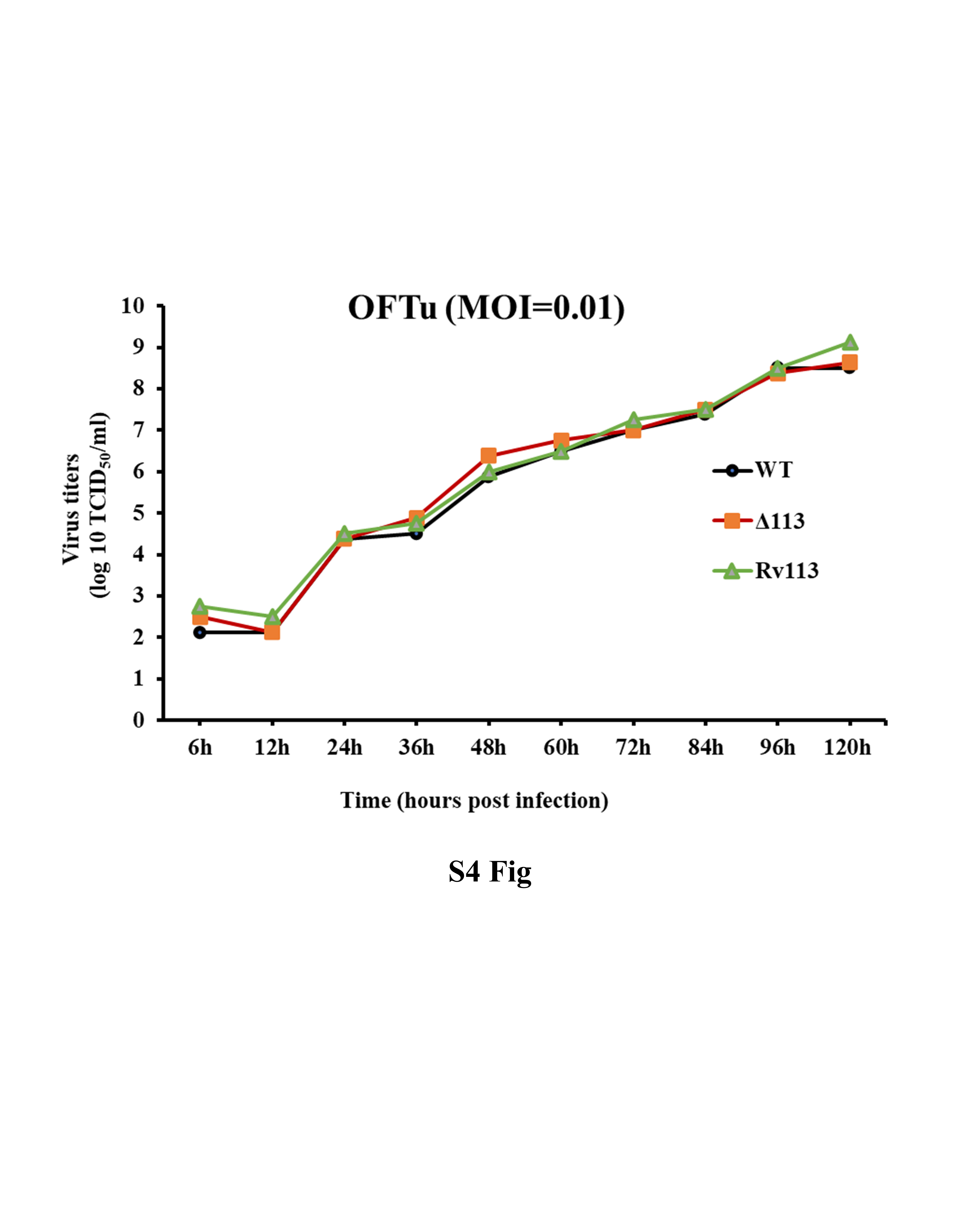

Supplement: S4 Fig — OFTu cells were infected with wildtype OV-IA82 (WT), ORFV113 deletion mutant OV-IA82Δ113 (Δ113) or revertant OV-IA82RV113Flag (RV113) viruses at MOI 0.01. Titers were determined at indicated times p.i. and expressed as TCID50/ml. Result is representative of two independent experiments. (TIF) [file ppat.1009971.s004.TIF]

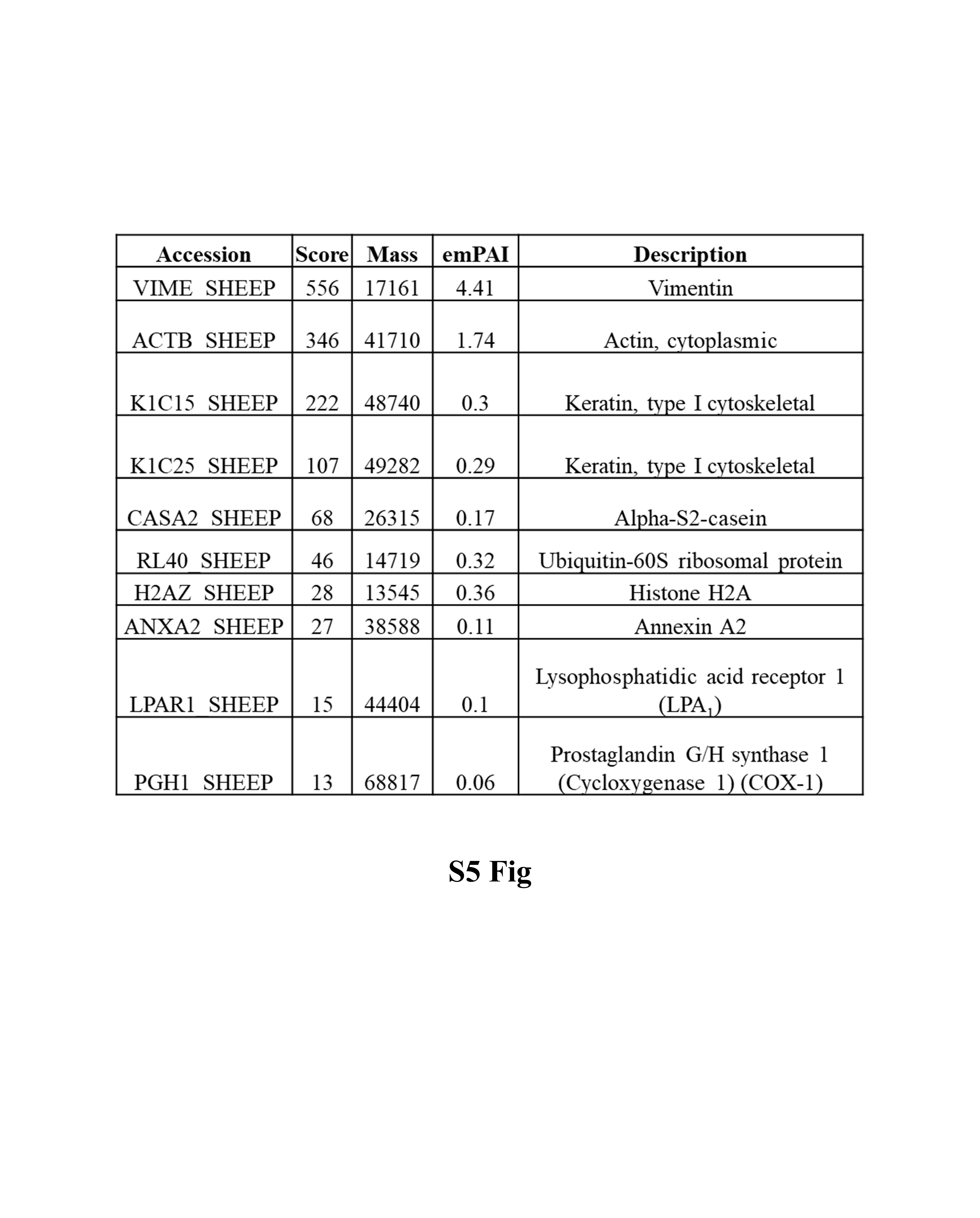

Supplement: S5 Fig — OFTu cells transfected with plasmid pCMV-ORFV113Flag was harvested at 24 h post transfection and total cell protein was extracted. Immunoprecipitation was performed using anti-flag antibody. LC-MS Mass Spectrometry was performed, and data were analysed using in house Mascot server. Cut off score was 3 at p<0.05. emPAI (Relative abundance) (TIF) [file ppat.1009971.s005.TIF]

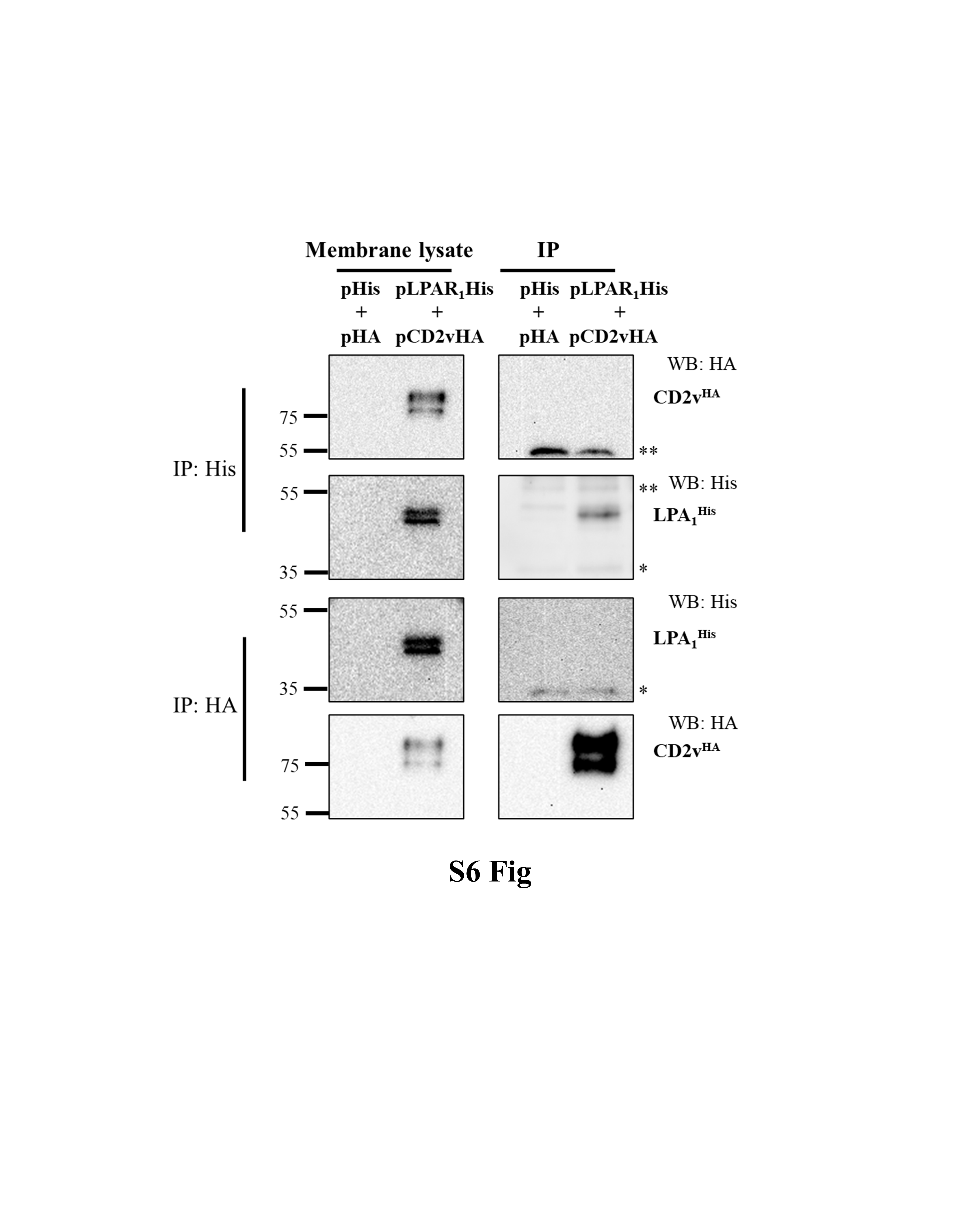

Supplement: S6 Fig — OFTu cells co-transfected with plasmids pcDNA3.1His (pHis)and pCMV-HA (pHA) (Empty plasmids) or pcDNA3.1-LPA1His (pLPA1His) and pCMV-CD2vHA (pCD2vHA) were harvested at 24 h post transfection and membrane fractions were extracted. Membrane lysates (left) and extracts immunoprecipitated with anti-His (upper panel) or anti-HA (lower panel) antibodies were analyzed by SDS-PAGE-Western blotting with antibodies directed against proteins indicated on the right. * and ** denote light and heavy chain of the IgG antibody, respectively. (TIF) [file ppat.1009971.s006.TIF]

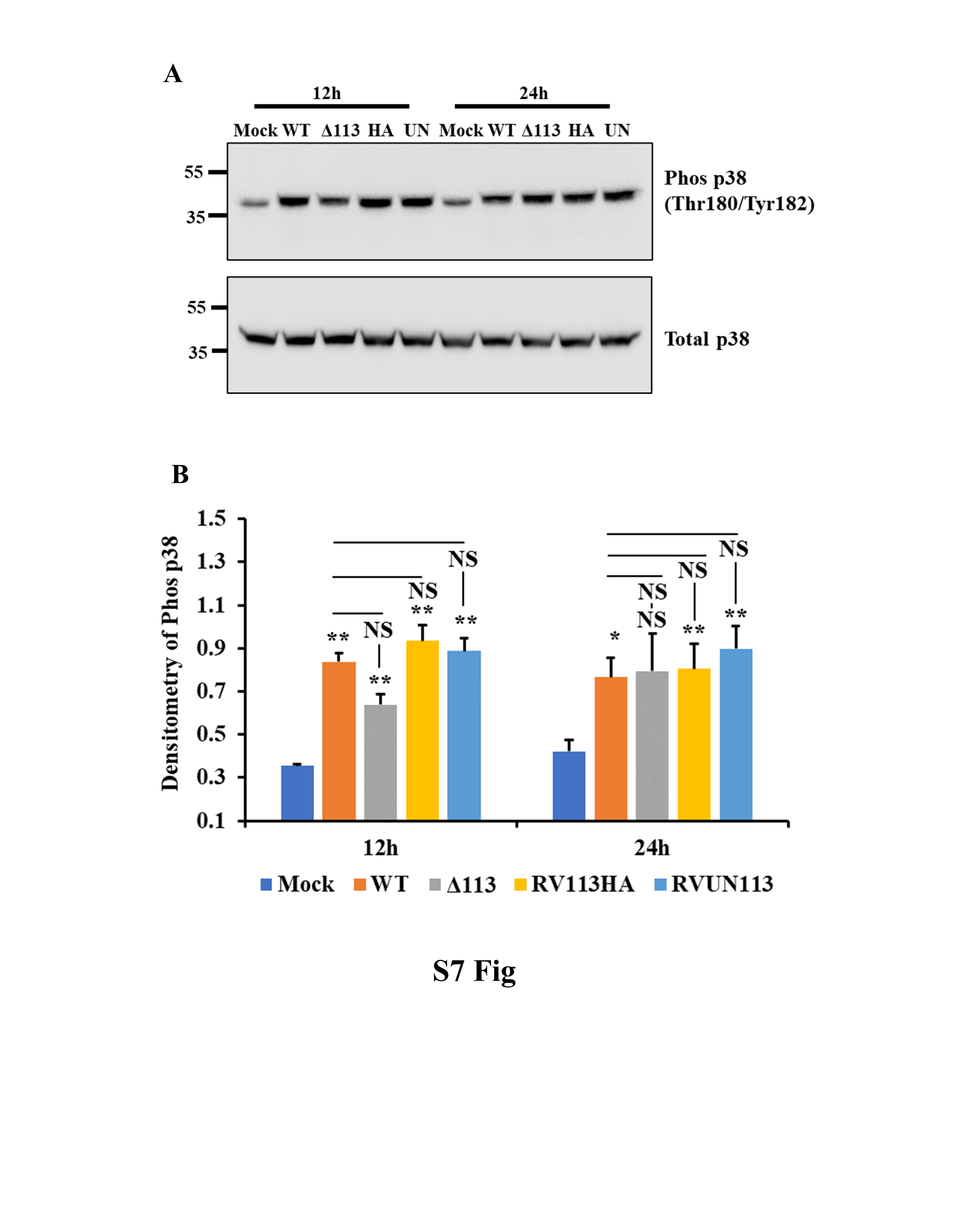

Supplement: S7 Fig — (A) OFTu cells were mock-infected or infected with OV-IA82 (WT), OV-IA82-Δ113 (Δ113), OV-IA82-RV113HA (HA) and OV-IA82-RV113 (UN) (MOI:10) for 12 and 24 h p.i. Total cell protein extracts were resolved by SDS-PAGE, blotted and incubated with antibodies against phos-p38 and total p38. (B) Densitometric analysis of bands corresponding to phosphorylated p38. Densitometry of phos-p38 bands were normalized to the loading control total p38. Results are the average of three independent experiments. Error bars represent mean±SD. Statistical analysis was performed using one-way ANOVA with post hoc Tukey test for multiple comparisons (*, P<0.05; **, P<0.01, NS: non-significant). * denote statistical significance compared to mock. Statistical comparison of a treatment to WT is shown with a line drawn over the respective treatment bar. (TIF) [file ppat.1009971.s007.TIF]

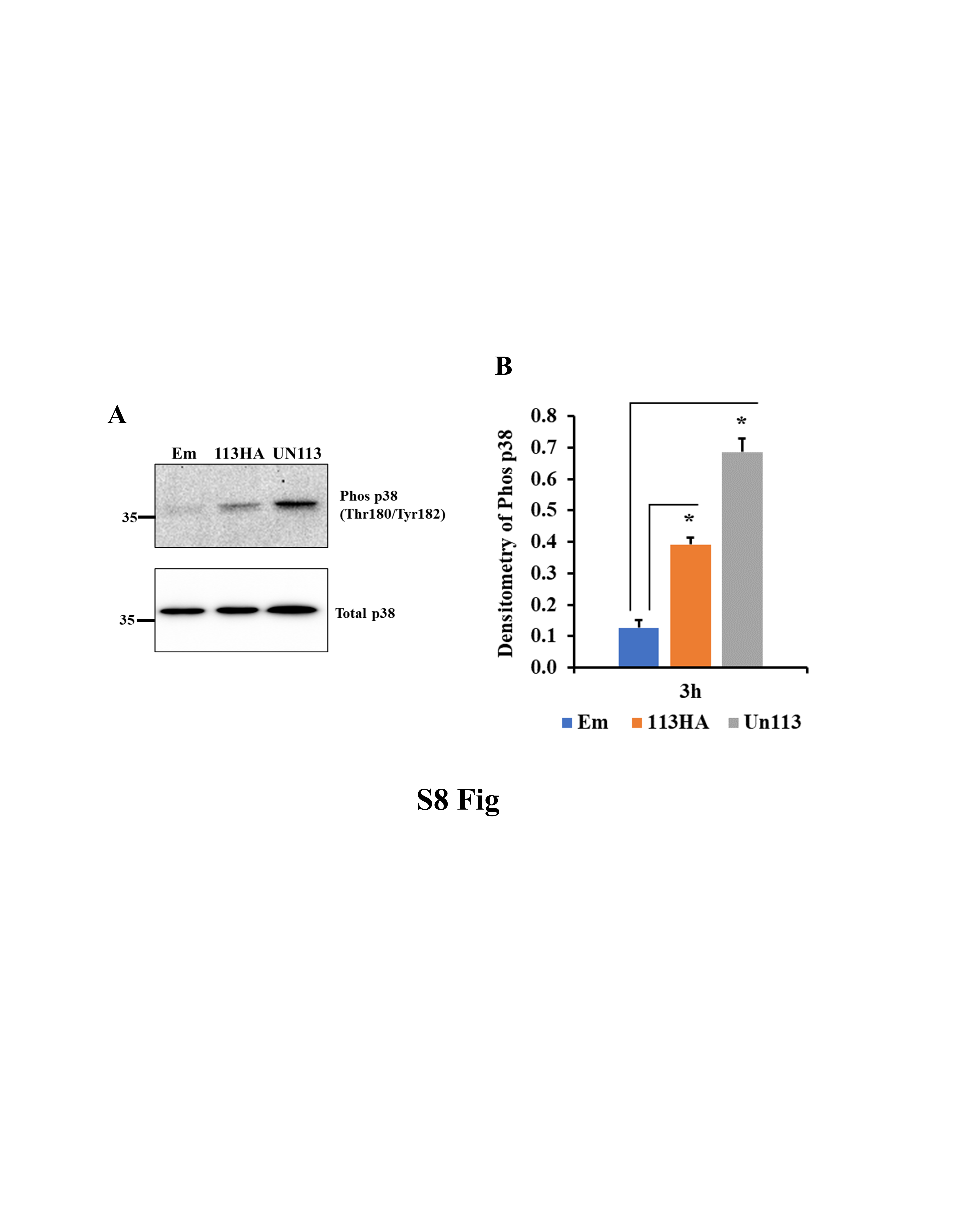

Supplement: S8 Fig — (A) Culture supernatants were harvested from OFTu cells transfected with pCMV-HA (Empty HA plasmid), pCMV-ORFV113HA (ORFV113HA plasmid) or pCMV-ORFV113 (Untagged ORFV113 plasmid) (UN113) at 24 h post transfection. Naive OFTu cells were then treated with respective clarified supernatants and harvested at 3 h post treatment. Total cell protein extracts were resolved by SDS-PAGE, blotted and incubated with antibodies against phos-p38 and total p38. (B) Densitometric analysis of bands corresponding to phosphorylated p38. Densitometry of phos-p38 bands were normalized to total p38. Results are the average of three independent experiments. Error bars represent mean±SD. Statistical analysis was performed using one-way ANOVA with post hoc Tukey test for multiple comparisons (*, P<0.05; **, P<0.01). (TIF) [file ppat.1009971.s008.TIF]
